# 6-Gingerol reduces *Pseudomonas aeruginosa* biofilm formation and virulence via quorum sensing inhibition

**DOI:** 10.1038/srep08656

**Published:** 2015-03-02

**Authors:** Han-Shin Kim, Sang-Hoon Lee, Youngjoo Byun, Hee-Deung Park

**Affiliations:** 1 School of Civil, Environmental and Architectural Engineering, Korea University, Anam-Dong, Seongbuk-Gu, Seoul 136-713, South Korea; 2 College of Pharmacy, Korea University, Sejong-ro 2511, Jochiwon-eup, Sejong, 339-700, South Korea

## Abstract

*Pseudomonas aeruginosa* is a well-known pathogenic bacterium that forms biofilms and produces virulence factors via quorum sensing (QS). Interfering with normal QS interactions between signal molecules and their cognate receptors is a developing strategy for attenuating its virulence. Here we tested the hypothesis that 6-gingerol, a pungent oil of fresh ginger, reduces biofilm formation and virulence by antagonistically binding to *P. aeruginosa* QS receptors. *In silico* studies demonstrated molecular binding occurs between 6-gingerol and the QS receptor LasR through hydrogen bonding and hydrophobic interactions. Experimentally 6-gingerol reduced biofilm formation, several virulence factors (e.g., exoprotease, rhamnolipid, and pyocyanin), and mice mortality. Further transcriptome analyses demonstrated that 6-gingerol successfully repressed QS-induced genes, specifically those related to the production of virulence factors. These results strongly support our hypothesis and offer insight into the molecular mechanism that caused QS gene repression.

P*seudomonas aeruginosa* is a notorious opportunistic and nosocomial pathogenic bacterium infecting immunocompromised patients^1^. It infects pulmonary and urinary tracts, burns, and wounds, sometimes resulting in serious health complications^2^,^3^. In particular, it is fatal to cystic fibrosis patients, the most common genetic disorder in Caucasians^4^, by forming mucoid in lung tissue leading to pneumonia^5^. Due to its metabolic versatility, *P. aeruginosa* is observed in diverse natural and man-made environments such as natural water bodies, soil, skin, and many medical devices^6^,^7^. *P. aeruginosa* can colonize on various surfaces by forming a biofilm in which bacterial cells stick together and are embedded within a self-produced extracellular polysaccharide matrix^8^. Biofilm cells of *P. aeruginosa* are reported to be more resistant to antibiotics and biocides than planktonic cells, which often cause difficulties in eradicating them from patients infected with the bacterium^9^. A means to control biofilm growth to more effectively treat *P. aeruginosa* infections is thus needed.

Quorum sensing (QS) is a bacterial communication system for coordinating group behaviors such as forming biofilms and producing virulence factors. QS is a signal and response based system dependent on population density. Chemical signal molecules called autoinducers (AIs) increase in concentration with population density and are received by transcriptional regulators that control gene expression^10^. Gram-negative bacteria including *P. aeruginosa* use *N*-acylated homoserine lactones (AHLs) as AI molecules. *P. aeruginosa* has three main QS systems: LasI-LasR, RhlI-RhlR, and PQS-MvfR. LasI produces an extracellular diffusible AHL signal molecule, *N*-(3-oxododecanoyl)-L-homoserine lactone (OdDHL) shown in Fig. 1a. OdDHL is recognized by the transcriptional regulator LasR which directs various gene expressions including genes affecting the RhlI-RhlR system. Likewise, RhlI produces the *N*-butyryl-L-homoserine lactone (BHL) signal molecule (Fig. 1a) which can bind to its cognate transcriptional regulator RhlR. LasR and RhlR transcriptional regulators are activated when sufficient levels of OdDHL and BHL are present resulting from a high population density of *P. aeruginosa* cells^11^,^12^. In the PQS-MvfR system, 2-heptyl-3-hydroxy-4(1H) quinolone (PQS) and its precursors bind to the transcriptional regulator MvfR and control the transcription of downstream targets^13^. More recently, 2-(2-hydroxyphenyl) thiazole-4-carbaldehyde (IQS) was identified as a new QS signal which is associated with phosphate-stress response in *P. aeruginosa*^14^. Furthermore, *cis*-unsaturated fatty acids known as diffusible signal factors (DSFs) are known to be involved in cell-cell signaling in biofilm formation, cell growth, and virulence factor production^15^. In *P. aeruginosa*, *cis*-2-decenoic acid is reported as a DSF inducing biofilm dispersion and biofilm formation inhibition^16^.

Many bacteria utilize QS for the production of biofilms and virulence factors during pathogenesis. Thus, the inhibition of QS is regarded as an attractive approach to control bacterial infection^17^,^18^. Several natural and synthetic anti-QS compounds have been introduced in previous studies. Extracts of various natural products (e.g., bean sprout, chamomile, carrot, and garlic)^19^ and essential oils of several plants (e.g., lavender, eucalyptus, and citrus)^20^ have shown anti-QS effects. Synthetic furanones and AHL analogs are well known man-made anti-QS compounds, used in laboratory studies only, effective at reducing the virulence of *P. aeruginosa*. Synthetic furanones and AHL analogs are assumed to competitively bind to *P. aeruginosa* LasR and/or RhlR, which inhibits the OdDHL and/or BHL from binding to the respective cognate transcriptional regulator^21^,^22^. However, no detailed competitive binding mechanism has been reported.

We previously reported on the anti-biofilm effect of ginger extract on *P. aeruginosa*^23^. Ginger (*Zingiber officinale*) rhizome contains various volatile oils (e.g., zingeberene, curcumene, and farnesense), non-volatile oils (e.g., gingerols, shogaols, paradols, and zingerone), and other ingredients (e.g., oleoresins, L-ascorbic acid, myricetin, fats, carbohydrates, vitamins, and minerals)^24^. Among the oils, 6-gingerol has been studied extensively. 6-Gingerol has been reported to possess various pharmacological properties such as anti-cancer effects^25^, anti-inflammatory effects, analgesic effects^26^, along with being cytotoxic and apoptotic to human promyelocytic leukemia (HL-60) cells^27^. In addition, a recent study reported on the anti-QS effects of 6-gingerol^28^, but did not include the anti-QS mechanism of 6-gingerol or the effect 6-gingerol on biofilm formation or virulence.

6-Gingerol contains a hydroxyl group at the 5-position, a carbonyl group at the 3-position, and a 4′-hydroxy-3′-methoxyphenyl group at the 1-position of its decane backbone structure (Fig. 1a). Similar to the OdDHL-based QS inhibitors^29^, 6-gingerol has a long alkyl chain which may provide the binding specificity for LasR and aromatic functionality which may characterize agonistic or antagonistic properties. We hypothesized that 6-gingerol might bind to the ligand-binding domain of LasR in a similar way to the natural ligand OdDHL.

The primary objective of this study was to test the hypothesis that 6-gingerol is an anti-QS molecule. The effect of 6-gingerol on biofilm formation and virulence in *P. aeruginosa* was evaluated. The potential of 6-gingerol to cause QS inhibition in *P. aeruginosa* was tested using both a molecular docking analysis comparing 6-gingerol and OdDHL and a QS competition assay using reporter strains. Furthermore, we identified 6-gingerol responsive *P. aeruginosa* genes using microarray and reverse transcription quantitative PCR (RT-qPCR) of genes within the QS regulon to gain insight into the molecular mechanisms of 6-gingerol QS inhibition.

## Results

### Effect of 6-gingerol on biofilm formation and growth

**Figure 1:**
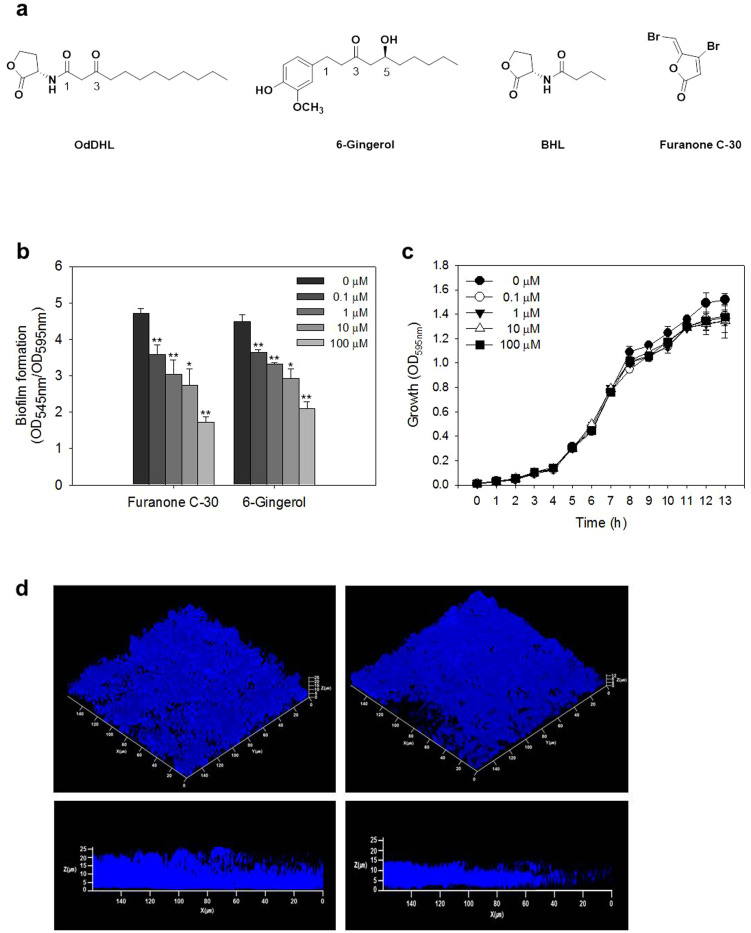
Effects of 6-gingerol on *P. aeruginosa* biofilm formation and growth. (a) Chemical structures of *N*-(3-oxododecanoyl)-L-homoserine lactone (OdDHL), 5-hydroxy-1-(4-hydroxy-3-methoxyphenyl)-3-decanone (6-gingerol), *N*-butyryl-L-homoserine lactone (BHL), and (Z-)-4-bromo-5-(bromomethylene)-2(5H)-furanone (furanone C-30). (b) Biofilm formation at different concentrations of 6-gingerol and furanone C-30 (0, 0.1, 1, 10, and 100 μM) for 24 h in microtiter plate. Error bars indicate the standard deviations of 8 measurements. **, P < 0.00001 versus the control. *, P < 0.0005 versus the control. (c) Growth at different concentrations of 6-gingerol (0, 1, 10 and 100 μM) for 13 h in flask. Error bars indicate the standard deviations of 3 measurements. (d) CLSM images of biofilms formed in drip-flow reactor for 24 h with 10 μM 6-gingerol (right) and without (left). The biofilm cells were stained with DAPI and observed under blue fluorescence light. Depth of the two corresponding biofilms was analyzed by Z-stack images (low). The same volume of DMSO was added to the “0 μM 6-gingerol” treatments as the 6-gingerol treatments.

*P. aeruginosa* biofilm formation with 6-gingerol was evaluated using a static biofilm assay. Biofilm formation was reduced 19–53% by 6-gingerol in a concentration-dependent manner as shown in Fig. 1b. Furanone C-30, the positive control of this experiment and a reported potent QS inhibitor^21^, also reduced biofilm formation by 24–63%, similar to 6-gingerol, at the same concentration range. These results demonstrated that 6-gingerol was as effective as a synthetic anti-QS compound, furanone C-30, in inhibiting biofilm formation.

The reduction of biofilm formation by 6-gingerol was also demonstrated in a continuous drip-flow reactor. Figure 1d shows Confocal Laser Scanning Microscope (CLSM) images of *P. aeruginosa* biofilms formed on a glass surface in the reactor. The biofilm formed with 10 μM 6-gingerol was observed to be shallower and less dense than the control biofilm. Based on Fig. 1d, typically, the height of the biofilm formed with 10 μM and 0 μM 6-gingerol was ~12 and ~25 μm, respectively.

The effect of 6-gingerol on *P. aeruginosa* growth was evaluated by monitoring the OD of batch cultures at 595 nm (Fig. 1c). The pattern of *P. aeruginosa* growth was not significantly different between the cultures with 0, 0.1 1, 10 and 100 μM 6-gingerol during the lag and exponential growth phases, but there was a slight inhibition of growth at the end of the exponential and stationary phases by adding 0.1–100 μM 6-gingerol to the culture.

### Involvement of 6-gingerol in QS inhibition

The possibility of QS inhibition by 6-gingerol was initially explored using an AHL-based *in vitro* QS competition assay using two genetically modified strains, *C. violaceum* CV026 and *A. tumefaciens* NT1. In this study, *C. violaceum* CV026 was used to test QS inhibition associated with BHL, whereas *A. tumefaciens* NT1 was used to test QS inhibition associated with OdDHL. The *C. violaceum* CV026 strain was designed to produce the AHL receptor CviR, which can sense exogenous AHLs having a shorter carbon chain length including BHL. The *A. tumefaciens* NT1 strain generates the AHL receptor TraR, for the detection of AHLs including OdDHL. Although these strains do not produce the exact LasR and RhlR receptors found in *P. aeruginosa*, the TraR and CviR receptors are considered adequate indicators of LasR and RhlR behavior, respectively.

**Figure 2:**
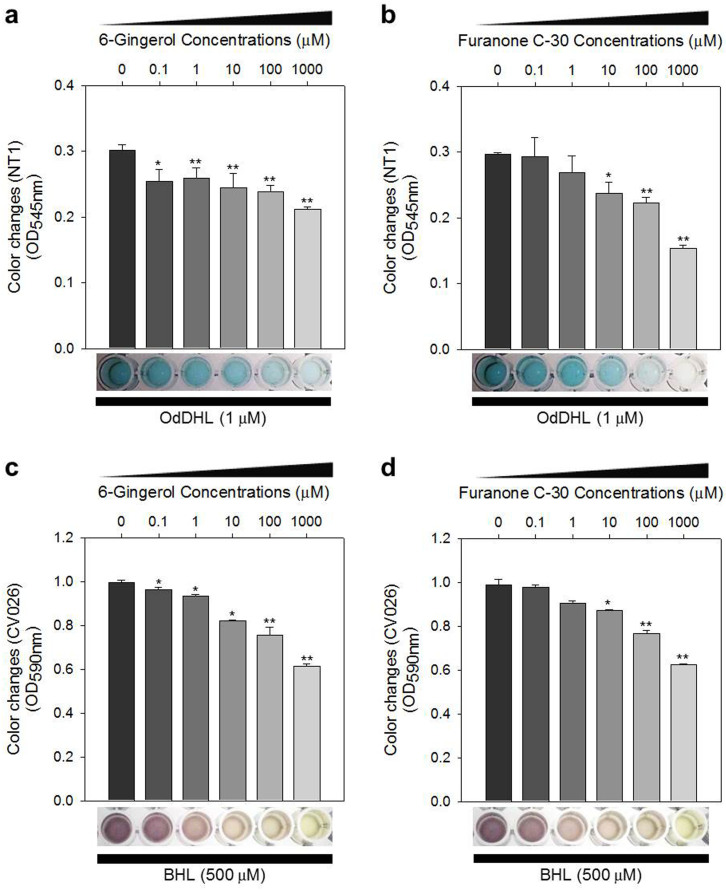
QS competition assay with 6-gingerol. *A. tumefaciens* NT1 and *C. violaceum* CV026 strains were used for assaying QS competition related to OdDHL and BHL, respectively. Color changes of NT1 or CV026 cultures at different 6-gingerol or furanone C-30 concentrations (0, 1, 10, 100, and 1000 μM). Color change was measured with OD at 545 nm for NT1 cultures and with OD at 590 nm for CV026 cultures, respectively. (a) 6-gingerol-OdDHL competition. (b) furanone C-30-OdDHL competition. (c) 6-gingerol-BHL competition. (d) furanone C-30-BHL competition. **, P < 0.0005 versus the control. *, P < 0.005 versus the control. The same volume of DMSO was added to the “0 μM 6-gingerol” treatments as the 6-gingerol treatments.

The assay was based on comparing the competitive binding of the AHL molecules of *P. aeruginosa* (e.g., BHL and OdDHL) and 6-gingerol to the AHL receptors in which the competition was measured colormetrically. Furanone C-30 was used as the positive control for the experiment (Figs. 2b and 2d). In this assay, the presence of color represents the binding of the AHL molecules to the AHL receptor, whereas the absence of color indicates the lack of AHL and AHL receptor binding. As shown in Fig. 2c, the CV026 cultures gradually decreased in purple pigment, violacein, with increasing 6-gingerol concentration (0.1–1,000 μM). Similar results were observed for the QS inhibition assays using NT1 in which cyan color was diminished by increasing 6-gingerol levels (Fig. 2a). These results demonstrate that 6-gingerol could have interfered with the interaction between the added AHLs (e.g., BHL and OdDHL) and their receptors (e.g., CviR for CV026 and TraR for NT1).

### Molecular docking analysis

The possibility of binding interference between OdDHL and its cognate signal receptor, LasR, by 6-gingerol was investigated by a molecular docking analysis. A PDB coordinate file of LasR (PDB ID: 2UVO) was downloaded and utilized for the docking studies. 2UVO is the X-ray structure of N-terminal OdDHL-binding domains of LasR in complex with OdDHL. To assess the docking accuracy of the CDOCKER module for the LasR receptor system, the root mean square distance (RMSD) value between the native crystal conformation and the best-scoring docked pose of OdDHL was measured. The RMSD value of the docked ligand pose relative to its crystal orientation was 0.9852 Å, indicating that the docking method can predict the correct orientation for LasR ligands with high accuracy. Due to the reproducible ability of the CDOCKER module to correctly dock the crystal ligand for the LasR receptor, docking studies of 6-gingerol were performed with the same default settings.

**Figure 3:**
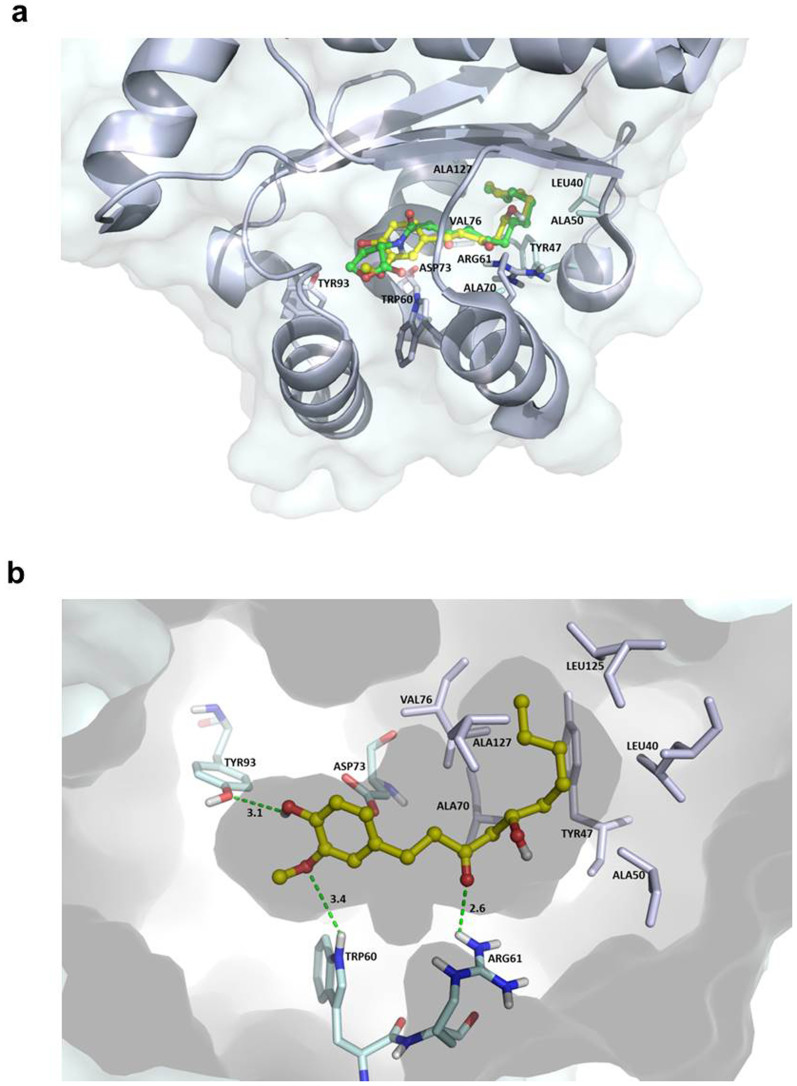
*In silico* molecular docking analysis. (a) Overlay of the crystal ligand OdDHL (green) with the docked pose of 6-gingerol (yellow) in LasR LBD domain (PDB ID: 2UV0). (b) Hydrogen-bonding interactions of 6-gingerol with LasR protein. Numbers indicate the distance in Å.

Figure 3 shows the overlay of the crystal orientation of OdDHL with the best-scoring docked 6-gingerol. The 3′-hydroxy-4′-methoxyphenyl moiety and the alkyl side chain of 6-gingerol overlapped with the homoserine ring and dodecanoyl group of OdDHL, respectively. The best-docked 6-gingerol fit inside the OdDHL binding site of the LasR protein. 6-Gingerol made hydrogen-bonding interactions with the Trp60, Arg61, and Tyr93 and hydrophobic interactions with Leu40, Tyr47, Ala50, Ala70, Val76, and Ala127. Trp60 and Arg61 were key amino acids to make hydrogen-bonding interactions with the X-ray crystal ligand OdDHL.

### Effects of 6-gingerol on virulence

**Figure 4:**
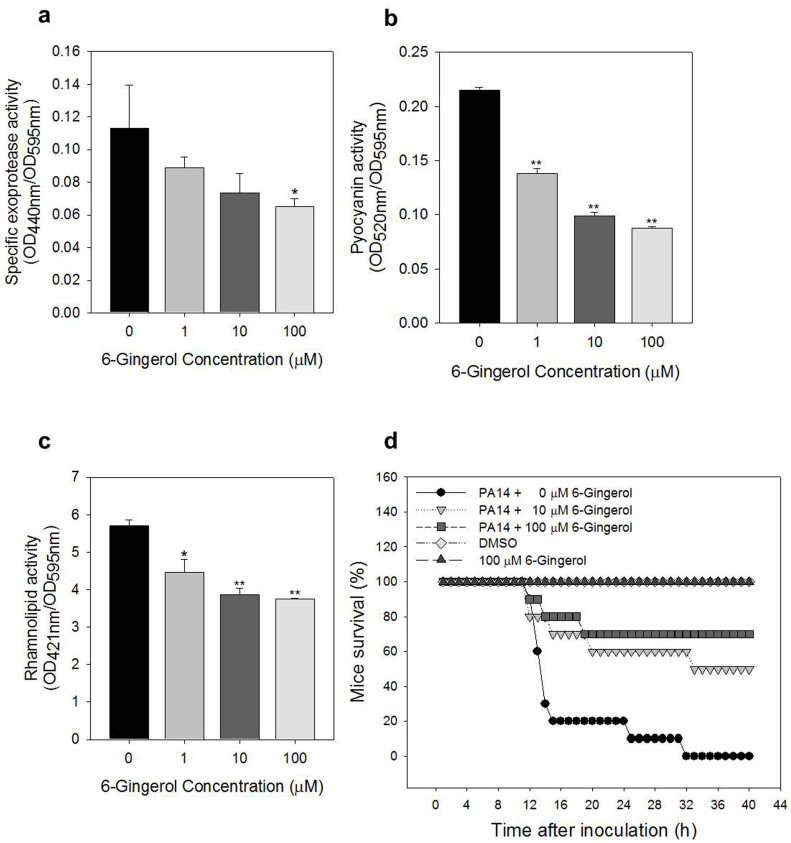
Effect of 6-gingerol on virulence. Activities of virulence factors at different 6-gingerol concentrations (0, 1, 10, and 100 μM) were analyzed. (a) Specific exoprotease activity. (b) Pyocyanin activity. (c) Rhamnolipid activity. Error bars indicate the standard deviations of 3 measurements. **, P < 0.00005 versus the control. *, P < 0.05 versus the control. (d) mice mortality was evaluated for the specific pathogen free ICR female mice injected with *P. aeruginosa* grown with 0, 10, and 100 μM, respectively. Mice injected with 1% DMSO and 100 μM 6-gingerol were used as negative controls. The same volume of DMSO was added to the “0 μM 6-gingerol” treatments as the 6-gingerol treatments.

Three virulence factors (exoprotease, pyocyanin, and rhamnolipid) and mice mortality were analyzed to assess the effects of 6-gingerol on virulence. Production of the three virulence factors was reduced by 6-gingerol in a concentration dependent manner (0, 1, 10, and 100 μM 6-gingerol added): 21–43% suppression for exoprotease, 36–60% suppression for pyocyanin, and 36–60% suppression for rhamnolipid (Figs. 4a, b, and c).

In the mice mortality study shown in Fig. 4d, mice injected with *P. aeruginosa* grown with no 6-gingerol started to die after 12 hrs of incubation, and all of them were dead after 31 hrs. Mice injected with *P. aeruginosa* grown with 6-gingerol showed significant improved survival rates. 50% and 70% of the mice injected with *P. aeruginosa* grown with 10 and 100 μM 6-gingerol, respectively, were alive at the end of the 40 hrs incubation period.

### Transcriptome analysis of *P. aeruginosa* biofilm cells treated with 6-gingerol

The transcriptome of *P. aeruginosa* biofilm cells was analyzed to identify the genes targeted by 6-gingerol and to investigate the molecular mechanism that reduces biofilm formation and virulence when 6-gingerol is added. An Affymetrix GeneChip *P. aeruginosa* DNA microarray was used to compare the gene expression between *P. aeruginosa* biofilm cells with and without 6-gingerol present. As shown in Supplementary data 1, 321 and 374 *P. aeruginosa* genes (out of 5,570 analyzed genes) were activated and repressed, respectively, by 6-gingerol based on a 1.5-fold cutoff. These genes were related to QS (7.2%), motility (2.0%), type III secretion (3.5%), ribosomal protein (3.2%), cell division (0.7%), cell wall synthase (1.3%), metabolism (8.3%), transcriptional regulator (5.9%), hypothetical protein (40.4%), and other genes (27.5%).

A total of 365 QS-related genes were defined based on the list of QS-inducible genes identified by Hentzer et al.^21^, Schuster et al.^30^, and Wagner et al.^31^. DNA microarray data showed that 41% of the QS-related genes were down-regulated in the biofilm cells treated with 10 μM 6-gingerol, and the inventory is described in Supplementary data 2. The genes involved in the production or binding of QS signal molecules (e.g., *lasI*, *lasR*, and *rhlI*) were not influenced by 6-gingerol (<1.5-fold repression), except for the repression of *rhlR* encoding a transcriptional regulator of several QS responsive genes such as *mvfR*, *rhlA*, *rhlB*, *rhlI*, and *rhlR*. Interestingly, the gene, *rsaL*, encoding the transcriptional regulator that can bind to the *lasI* promoter and repress its transcription^32^ was observed to be overexpressed.

**Figure 5:**
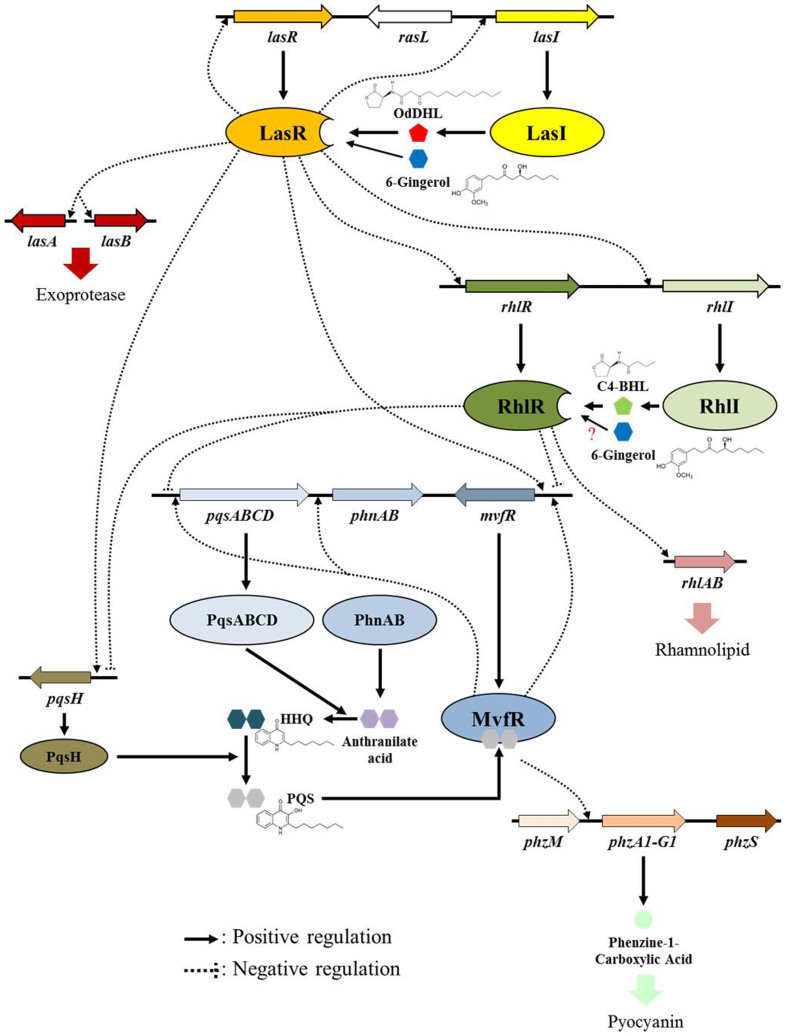
A schematic depicting QS system and virulence factor production gene expression alteration caused by 6-gingerol. Genes within the LasI-LasR and RhlI-RhlR QS systems and those related to the production of virulence factors (e.g., exoprotease, rhamnolipid, and pyocyanin) are focused on in this schematic. Positive and negative regulations of the gene expression are indicated as arrows (↑) and bars (⊥) signs, respectively.

The genes involved in the production of virulence factors such as rhamnolipid, elastase, and pyocyanin were highly repressed. These genes are shown in the flow chart in Fig. 5 and include the *rhlAB* operon encoding the rhamnosyltransferase chain, the *lasB* gene encoding elastase, and the *phzC-G* operon encoding phenazine. The genes involved in the synthesis of the MexGHI-OpmD efflux pump were also repressed. The efflux pump is known to be activated by QS and to play an important role in active AHL efflux, antibiotic resistance, and promoting virulence^33^. These genes include *mexI* encoding a multidrug efflux transporter, *mexH* encoding a multidrug efflux membrane fusion protein precursor, and *opmD* encoding an outer membrane efflux protein precursor.

The *Pseudomonas* quinolone signal (PQS) operons, *phnAB* and *pqsB*-E, were also repressed by 6-gingerol. The PQS molecule, 2-heptyl-3-hydroxy-4-quinolone, is reported to bind to its cognate receptor, MvfR, and activate the expression of the *phnAB* and *pqsA-E* operon and increase PQS and pyocyanin production^34^. Transcription of type III export protein related genes, the *pscD-K* operon, and *exoT* encoding type III effector protein and exoenzyme T were also repressed. These genes are essential for the pathogenicity of *P. aeruginosa* and are controlled by QS systems^31^.

Our DNA microarray data was analyzed based on the *P. aeruginosa* QS regulon which was defined by Givskov research group^35^,^36^ (Supplementary data 3). The QS regulon consists of 174 genes which can be classified into four groups: genes down-regulated by *lasR* and *lasRrhlR* mutants (group A), by *rhlR* and *lasRrhlR* mutants (group B), by *lasR*, *rhlR*, and *lasRrhlR* mutants (group C), and by *lasRrhlR* mutant (group D). Within the QS regulation, 32%, 38%, and 22% of group A, C, and D genes were down-regulated by 6-gingerol, respectively. However, only 13% of the group B genes were down-regulated, indicating 6-gingerol partially affected RhlR-controlled genes. This result suggests that *P. aeruginosa* QS was inhibited by 6-gingerol via LasR-controlled genes, similar to the antibiotics disrupting flux of OdDHL across cell membrane^35^.

**Figure 6:**
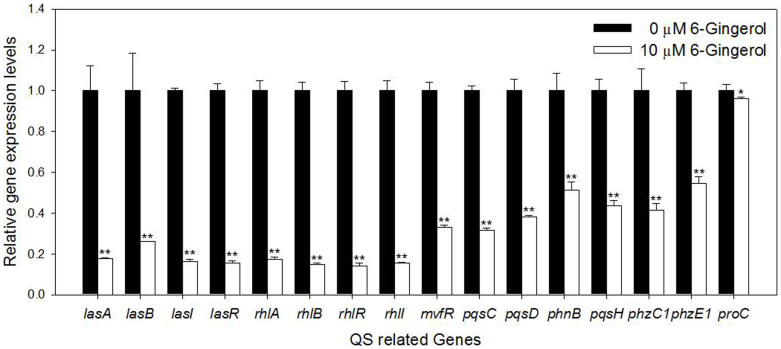
Effect of 6-gingerol on the expression of QS-inducible genes assessed by RT-qPCR. The relative magnitude of gene expression level was defined as the copy number of cDNA of each gene in the biofilm cells normalized by the copy number of cDNA of the corresponding gene in biofilm cells without 6-gingerol. Error bars indicate the standard deviations of 3 measurements. **, P < 0.005 versus the control. *, P < 0.05 versus the control. The same volume of DMSO was added to the “0 μM 6-gingerol” treatments as the 6-gingerol treatments.

The down expression of QS-related genes in the microarray was verified using RT-qPCR. To this end, 15 QS-inducible genes (*lasA*, *lasB*, *lasI*, *lasR*, *rhlA*, *rhlB*, *rhlR*, *rhlI*, *mvfR*, *pqsC*, *pqsD*, *phnB*, *pqsH*, *phzC1*, and *phzE1*) were selected, and the expressions of these genes were quantified in *P. aeruginosa* biofilm cells treated with 0 or 10 μM 6-gingerol. As shown in Fig. 6, all of the selected QS-inducible genes were significantly down expressed in the biofilm cells with 6-gingerol (45–86% compared with control), although *lasI*, *lasR*, *rhlI*, *mvfR*, and *pqsH* were not repressed in the analysis of DNA microarray. The expression of *lasA*, *lasB*, *lasI*, *lasR*, *rhlA*, *rhlB*, *rhlR*, and *rhlI* genes were comparatively more down-regulated (74–86%) than *mvfR*, *pqsC*, *pqsD*, *phnB*, *pqsH*, *phzC1*, and *phzE1* genes (45–68%). The expression of the *proC* housekeeping gene was not strongly affected by 6-gingerol.

## Discussion

This study clearly demonstrated that 6-gingerol inhibited *P. aeruginosa* biofilm formation in a concentration-dependent manner without a significant effect on its growth. Biofilms formed on glass surfaces with 6-gingerol were shallower and less dense than those without. In addition, 6-gingerol reduced the production of extracellular virulence factors such as exoprotease and pyocyanin and significantly decreased the mice mortality rate of *P. aeruginosa*.

Based on Fig. 5, these effects of 6-gingerol on *P. aeruginosa* begin with 6-gingerol binding with the receptor LasR, which was evidenced by the molecular docking analysis. *In silico* docking studies of 6-gingerol with LasR showed the potential binding mode of 6-gingerol to the active site of the ligand-binding domain. The binding mode of 6-gingerol was similar to that of the crystal ligand OdDHL, by a combination of hydrogen bonds and hydrophobic interactions. Recent comprehensive structure-activity relationship studies of OdDHL analogs showed that the replacement of the homoserine lactone moiety (present in OdDHL) with a substituted aromatic system changed the QS agonistic properties into antagonistic properties^37^,^38^,^39^. Thus, the substituted phenyl ring of 6-gingerol might contribute to its antagonistic properties, while the long alkyl chain and 3-oxo function provides the specificity for LasR receptor. In particular, the phenolic hydroxyl group of 6-gingerol made direct hydrogen bonding interactions with Tyr93, which was not observed in the crystal LasR and OdDHL complex.

The possibility of 6-gingerol's competition with OdDHL for binding to LasR was also supported by the NT1-based QS competition assay in which the effect of exogenous OdDHL decreased by increasing 6-gingerol concentration (Fig. 2). However, the colormetric results displayed in Fig. 2a were not as conclusive as expected given the molecular docking results and the measured biofilm depth and virulence factor reduction from Figs. 1b and 4, respectively. As mentioned previously, a proxy receptor protein, TraR, was used instead of LasR for the QS competition assay, which may account for the lack of a substantial concentration based reduction of OdDHL and TraR binding with 6-gingerol. To obtain direct evidence that LasR is the target of 6-gingerol, we also evaluated 6-gingerol inhibition of biofilm formation in *P. aeruginosa* transformed by a plasmid overexpressing LasR (refer to supplementary information for detailed experimental procedures). The transformed *P. aeruginosa* did not display any biofilm formation inhibition with the addition of 6-gingerol (0–100 μM), suggesting that 6-gingerol targeted LasR in *P. aeruginosa* (Supplementary figure 1).

The binding of 6-gingerol to the LasR signal receptor causes it to lose its function as a transcriptional activator. The down expression of *lasA*, *lasB*, *lasI*, *lasR*, *rhlA*, *rhlB*, *rhlR*, *rhlI*, *mvfR*, *pqsC*, *pqsD*, *phnB*, *pqsH*, *phzC1*, and *phzE1* in the RT-qPCR results support our speculation (Fig. 6). Down expression of these genes may interfere with normal production of several virulence factors including exoprotease, rhamnolipid, and pyocyanin via the actions of the *lasAB*, *rhlAB*, *phzA1-G1* operons, respectively. Reduced production of these virulence factors by 6-gingerol (Fig. 4) support our speculation.

6-Gingerol and LasR binding also appears to interfere with normal QS circuitry in *P. aeruginosa* via the molecular mechanism shown in Fig. 5. In a high cell density environment, normal QS circuitry of *P. aeruginosa* is initiated by binding OdDHL to its cognate signal receptor, LasR. The OdDHL and LasR complex acts as transcriptional activator and enhances the expression of several genes within QS regulon including the *lasA*, *lasB*, *lasI*, *lasR*, *rhlI*, *rhlR*, *mvfR*, and *pqsH* genes. The expression of *rhlR* activates the production of the BHL signal receptor, RhlR. The BHL and RhlR complex is the transcriptional activator for the *rhlAB* operon and the transcriptional repressor for the *mvfR* gene. Therefore, binding of 6-gingerol to the LasR signal receptor may cause a decrease in RhlR production that could result in a decrease in the *rhlAB* operon and *rhlI* production while removing transcriptional repression for *mvfR*.

The expression of *mvfR* turns on the cascade of genes (e.g., *phnAB*, *pqsA-E*, *phzA1-G1* operons) involved in the PQS system^40^ and pyocyanin production^41^. The OdDHL-LasR and BHL-RhlR complexes provide opposing effects on *mvfR* gene expression. The OdDHL-LasR complex acts as an activator for the *mvfR* gene, while the BHL-RhlR complex provides suppression of the gene^12^,^42^. These contrary effects may explain why the genes associated with MvfR in Fig. 6 (*mvfR*, *pqsC*, *pqsD*, *pqsH*, *phnB*, *phzC1*, and *phzE1*) are not as repressed as the other genes evaluated when exposed to 6-gingerol. They are repressed in respect to the control, likely due to less OdDHL-LasR complex activating the *mvfR* expression, but a decrease in the BHL-RhlR complex would also decrease gene suppression.

This hypothesis is supported by Choi et al.^43^ who studied *pqsA-E/phnAB* genes expression using RT-qPCR in *P. aeruginosa* *rhlR* and *lasRrhlR* mutants. They observed that expression of the genes was highly activated in the *rhlR* mutant and deduced that the result was caused by derepression of the genes. They also detected inactivation of the genes in *lasRrhlR* mutants, demonstrating a requirement of LasR in activating the genes.

Along with reducing the production of RhlR, 6-gingerol may also directly compete with BHL in binding to its cognate signal receptor, RhlR. Although a molecular docking study was not tried to test this possibility due to unavailability of the RhlR crystal structure, a QS competition assay using *C. violaceum* CV026 demonstrated its possibility (Fig. 2c). Both microarray and RT-qPCR results demonstrating the repression of *rhlI* and the *rhlAB* operon appear to support this speculation. However, the expression of these genes can also be repressed by impacting the LasI-LasR network, as shown in Fig. 5. As 6-gingerol shares some structural similarities with 2-heptyl-3-hydroxy-4(1H)-quinolone (PQS), a signal molecule of the PQS-MvfR system, it might compete with PQS to bind to MvfR. However, this study focused on the biochemical activity of 6-gingerol in relation to LasR of *P. aeruginosa*.

The significance of this study is the finding that 6-gingerol can reduce *P. aeruginosa* biofilm formation and virulence, which is crucial to increase antibiotic effectiveness and reduce the pathogenicity of *P. aeruginosa.* Furthermore, this study provides insight into the molecular mechanism of the QS inhibitory effects of 6-gingerol in *P. aeruginosa* via competitive binding to cognate receptors.

Ginger has been used as a culinary and medicinal herb for several thousands and is therefore considered a safe material. 6-Gingerol is a non-volatile oil constituting 1–3 wt. % of fresh ginger, which can be available readily with little cost. Hence, 6-gingerol presents a possibility for use as a safe material for combating *P. aeruginosa* biofilm growth in healthcare environments or as a medication for treating *P. aeruginosa* infections in the future.

## Methods

### Chemicals, bacterial strain, and growth measurement

6-Gingerol (5-hydroxy-1-(4-hydroxy-3-methoxyphenyl)-3-decanone) and furanone C-30 ((Z-)-4-bromo-5-(bromomethylene)-2(5H)-furanone), a positive control of QS inhibition, were purchased from Sigma Aldrich (St. Louis, MO, USA). The two chemicals were dissolved in dimethyl sulfoxide (DMSO) (Carl Roth, Karlsruhe, Germany). *P. aeruginosa* PA14 strain was used to evaluate the effect of 6-gingerol and furanone C-30. To test the growth inhibitory effect of 6-gingerol, *P. aeruginosa* was grown in AB medium^23^ with varying concentrations of 6-gingerol (0, 1, 10 and 100 μM) using a shaking incubator at 37°C at 250 rpm for 13 h. Growth was evaluated by measuring optical density (OD) at 595 nm using a UVmini-1240 spectrophotometer (Shimadzu, Kyoto, Japan) in triplicate samples collected hourly.

### Static biofilm formation assay

*P. aeruginosa* biofilm was formed in a TPP® 96-well polystyrene microtiter plate (Sigma Aldrich). Culture of *P. aeruginosa* (OD at 595 nm = 1.5) was diluted in fresh AB medium (1:20) containing either 6-gingerol or furanone C-30 (0–100 μM). The dilutions were aliquoted into the plate and were incubated at 37°C for 24 h without agitation. Following the 24 h incubation period, the OD of the suspended cells within each sample well was measured at 595 nm using an iMark microplate reader (BioRad, Richmond, CA, USA). The biofilm cells attached to the sample well surface were then stained using crystal violet for 30 min. Stained cells were washed with deionized water to remove unbound crystal violet and bounded crystal violet was eluted in 100% ethanol. The OD of the eluted ethanol samples were measured at 545 nm using an iMark microplate reader. For quantifying biofilm formation, the OD value at 545 nm was normalized by the OD value at 595 nm.

### Continuous-flow biofilm formation assay

*P. aeruginosa* biofilms were formed in a drip-flow biofilm reactor (DFR-110, BioSurface, MT, USA). Glass slides were dipped into a petri dish containing 2 ml of *P. aeruginosa* culture (OD at 595 nm = 1.5) and 18 ml of fresh AB medium to attach cells onto the slides and were incubated at 37°C for 24 h. The slides were then inserted into the drip-flow reactor system. Fresh AB medium with either 0 or 10 μM 6-gingerol was continuously fed into the reactor using a peristaltic pump (Masterflex C/L tubing pumps, Cole-Parmer, IL, USA) at 50 ml**·**h^−1^. The reactor operated at 37°C for 24 h. After stopping the feed, the suspended cells on the slide were carefully removed with phosphate-buffered saline (pH 7.2). The biofilm cells were stained with DAPI solution (Carl Roth) for 20 min. The biofilm cells were then observed using CLSM (Carl Zeiss LSM700, Jena, Germany) based on a previous study^23^. Confocal images of DAPI-stained biofilm cells were observed under blue fluorescence light (excitation wavelength: 350 nm, emission wavelength: 470 nm) using a 40× objective lens to evaluate the height and density of the biofilms (C-Apochromat 40×/1.20 W Korr M27, Carl Zeiss). The observed CLSM images were analyzed by the Zen 2011 program (Carl Zeiss).

### QS competition assay

An AHL-based QS competition assay was performed using the two reporter bacterial strains, *Chromobacterium violaceum* CV026^44^ and *Agrobacterium tumefaciens* NT1^45^, based on a previously study^23^. The *C. violaceum* CV026 strain was designed to produce the AHL receptor CviR, which can sense exogenous AHLs having a shorter carbon chain length including BHL. The *A. tumefaciens* NT1 strain generates the AHL receptor TraR, for the detection of AHLs including OdDHL. Although these strains do not produce the exact LasR and RhlR receptors found in *P. aeruginosa*, the TraR and CviR receptors are considered adequate indicators of LasR and RhlR behavior, respectively. 1 ml of overnight culture of either *C. violaceum* CV026 or *A. tumefaciens* NT1 strains were aliquoted into 15 ml conical tubes and the *A. tumefaciens* NT1 culture samples had 5 μL X-gal (100 μg/mL) added. The *C. violaceum* CV026 and *A. tumefaciens* NT1 samples then had either 1 μL of 500 mM BHL (BNPHARM, Daejeon, South Korea) or 1 mM OdDHL (Sigma Aldrich) added, respectively. Finally, either 10 μL of 6-gingerol (0–1000 μM) or furanone C-30 (0–1000 μM) was added to each sample. The cultures were then incubated at 30°C for 24 hr. QS competition was assayed by measuring OD at 590 nm and 545 nm for *C. violaceum* CV026 and *A. tumefaciens* NT1, respectively, using the spectrophotometer.

### Total RNA extraction

Total RNA was extracted from *P. aeruginosa* biofilm cells using the TRI REAGENT (Molecular Research Center, OH, USA) following the manufacturer's instruction. Biofilm cells were grown in 3 ml AB medium containing either 0 or 10 μM 6-gingerol at 37°C for 24 h in borosilicate bottles without agitation. Biofilm cells were collected into 1.5 ml micro-tubes, and then 1 ml of TRI REAGENT was added for homogenization. Harvested biofilm cells were centrifuged at 12,000 g at 4°C for 10 min to remove insoluble material such as extracellular membranes, polysaccharides, and high molecular mass DNA. Supernatants were mixed with 0.2 ml 99.9% chloroform (Carl Roth), and centrifuged at 12,000 g at 4°C for 15 min. Supernatant was collected into a new 1.5 ml micro-tube and 0.5 ml of 100% isopropyl alcohol (Merck, NJ, USA) was added. The mixture was then centrifuged at 12,000 g at 4°C for 10 min to form pellets. The pellets were washed with 1 ml of 75% ethanol and centrifuged at 7,500 g at 4°C for 5 min. Washed pellets were dissolved in nuclease-free water, and the quantity of total RNA was measured by an Agilent Bioanalyzer 2100 (Agilent Technologies, CA, USA).

### DNA microarray analysis

Differential gene expression of *P. aeruginosa* biofilm cells caused by 6-gingerol was analyzed using GeneChip *P. aeruginosa* Genome Array (900339, Affymetrix, CA, USA). A PAO1 chip has been used to analyze both PAO1 and PA14 transcriptome because of the high similarity of genomes between the two strains^46^. All of the procedures followed the manufacturer's protocols (Affymetrix) and were conducted at Seoulin Bioscience (Seongnam, South Korea). Briefly, total RNA (10 μg) was converted into cDNA using random primers, and the cDNA was fragmented using DNase I and biotinylated using terminal transferase. Biotinylated cDNA (5 μg) was then hybridized for 16 hr at 45°C on the GeneChip. An Affymetrix Fluidics Station 450 (Affymetrix) was used to wash and stain the GeneChip, and Affymetrix GeneChip Scanner 3000 7G (Affymetrix) was used to read the hybridization signals of the GeneChip. The Robust Multi-chip Average (RMA) algorithm was used for normalizing probe-level intensity^47^.

Microarray Suite version 5.0 (Affymetrix) was used to analyze GeneChip data using default analysis settings and global scaling. The differentially expressed genes were selected based on greater than 1.5-fold repression or induction by 6-gingerol.

### RT-qPCR

RT-qPCR was performed to quantify and compare the levels of gene expression for 15 *P. aeruginosa* QS genes when exposed to 6-gingerol. The primer sets for the genes were designed using Primer 3 version 0.4.0 (http://frodo.wi.mit.edu/) (Supplementary data 4). Utility of the designed primer sets was evaluated by measuring the length of the PCR amplicons in agarose gel and by sequencing them using a standard dideoxy method. RT-qPCR was performed using the Bio-Rad CFX-96 real time system (Bio-Rad, Hercules, CA, USA). The reaction mixture consisted of the following reagents: 10 μL SYBR Premix Ex Taq™ (Takara, Shiga, Japan), 0.8 μL each of the forward and reverse primers (10 μM), 0.4 μL of 50 X ROX™ Reference Dye I, 2 μL template RNA, and RNase free water to generate a 20 μL final volume. The thermal profile of the RT-qPCR for each of the target genes was as follows: initial denaturation at 95°C for 10 seconds (s), followed by 40 cycles of denaturation at 95°C for 10 s, annealing at 60°C for 10 s, and extension at 63°C for 34 s. Fluorescent signal intensity was collected at the end of the extension step. At the end of each run, a dissociation protocol (95°C for 15 s, 60°C for 1 min, and 95°C for 15 s) was performed to ensure that nonspecific amplicons were absent.

### Analysis of the production of virulence factors

Overnight culture of *P. aeruginosa* was inoculated in AB medium with or without 6-gingerol (0–100 μM) in 50 ml conical tubes and incubated using a shaking incubator at 250 rpm at 37°C for 24 hrs. The culture was then centrifuged at 12,000 g at 4°C for 5 min. For assaying exoprotease activity, 150 μL supernatant was mixed with 250 μL of 0.2% azocasein solution which was prepared by dissolving 0.1 g azocasein (Sigma Aldrich) in 50 ml of 50 mM Tris/HCl. The mixture was incubated at 4°C for 4 h without agitation. After reaction, 1.2 ml of 10% trichloroacetic acid (Sigma Aldrich) was added to the mixture and incubated at room temperature for 15 min. The mixture was then centrifuged at 10,000 rpm for 10 min. Supernatant was mixed in 1.4 ml of 1 M NaOH, and exoprotease activity was estimated by measuring OD at 595 nm in a spectrophotometer^48^. For assaying pyocyanin activity, crude pyocyanin was initially extracted by mixing 5 ml supernatant of the trichloroacetic acid containing samples with 3 ml of 100% chloroform. 1 ml of 0.2 N HCl was then added to the mixture to re-extract pyocyanin. Pyocyanin activity was estimated by measuring OD at 520 nm in a spectrophotometer^49^. For assaying rhamnolipid activity, crude rhamnolipid was initially extracted twice by mixing 500 μL cell supernatant of overnight culture with 1 ml of 100% diethyl ether (JUNSEI, Tokyo, Japan). The ether fraction was evaporated to dryness. The dry sample was eluted in 500 μL deionized water, and 100 μL of the elution was mixed with 900 μL Orcinal solution (0.19% Orcinal (Sigma Aldrich) in 53% H_2_SO_4_). The mixture was boiled for 30 min, and then cooled at room temperature for 15 min. Rhamnolipid activity was estimated by measuring OD at 421 nm in a spectrophotometer^50^. Exoprotease, pyocyanin, and rhamnolipid activity was normalized using the cell culture OD at 595 nm.

### Mice mortality test

Overnight culture of *P. aeruginosa* was inoculated in LB medium with 6-gingerol (0, 10, and 100 μM) using a shaking incubator at 250 rpm at 37°C for 7 hr. 1 ml of the culture (OD at 595 nm = 1) was harvested by centrifugation at 12,000 g at 4°C for 10 min. The pellet was resuspended in 2 ml PBS by vortexing. 100 μL of the suspended cells, equivalent to 2.5 × 10^7^ cells, were injected into the abdominal cavity of three-week-old specific pathogen-free ICR female mice (CrljOri:CD1[ICR], OrientBio, Seongnam, South Korea). Mortality was observed during a 40 hr period for ten mice for each treatment. 1% DMSO and 100 μM 6-gingerol in PBS was injected into mice as negative controls (3 mice each).

### Statistical analysis

Statistically analyzed *P*-values were estimated by a student's *t*-test (Exel software, Microsoft, Redmond, WA, USA).

### *In silico* docking study of 6-gingerol with LasR

6-Gingerol and OdDHL were drawn in ChemBioDraw (ver.11.0.1), transferred to Chem3D Pro (ver.11.0.1) to generate 3D structures, and saved as a mol file. The generated mol file was transferred to Discovery Studio 3.0 (Accelrys Inc., San Diego, USA). The process of ligand preparation and optimization was performed by Prepare Ligands Module, the protocol of Discovery Studio 3.0. The prepared ligands were converted to SD file format. The LasR protein structure in PDB format was downloaded from the RCSB Protein Data Bank (http://www.pdb.org). The LasR X-ray crystal structure with 1.80 Å resolution (PDB ID: 2UV0) was used for the docking studies^51^. Before conducting the docking procedure, the original crystal ligand OdDHL and water molecules were removed from the protein-ligand complexes and side chain bumps of amino acid residues were fixed. Hydrogen atoms were added by application of CHARMm Force Field, and Momany-Rone partial charge settings were used as default settings in Discovery Studio 3.0. The ligand binding site was extracted from PDB Site Records and defined as Active Site 1. CDOCKER Module of Discovery Studio 3.0 was used to perform the docking studies of 6-gingerol and OdDHL with LasR. The number of generated poses was set at 50 for each ligand and default settings were employed for other parameters.

## Author Contributions

H.S.K., Y.B. and H.D.P. conceived and designed the experiments. HSK performed most of the experiments. S.H.L. and Y.B. performed the RT-qPCR and *in silico* docking analyses, respectively. H.S.K., Y.B. and H.D.P. analyzed the experimental data and wrote the manuscript.

## Supplementary Material

Supplementary Information Supplementary Information
