# Supplementary material for: 6-Gingerol reduces Pseudomonas aeruginosa biofilm formation and virulence via quorum sensing inhibition
Source: Sci Rep. 2015 Mar 2;5:8656. doi: 10.1038/srep08656 (PMC4345325; doi:10.1038/srep08656)
Supplement: Supplementary Information [file srep08656-s1.pdf]

Supplementary information

6-Gingerol reduces *Pseudomonas aeruginosa* biofilm formation and virulence via quorum sensing inhibition

Han-Shin Kim[1], Sang-Hoon Lee[1], Youngjoo Byun[2*] and Hee-Deung Park[1*]

1-School of Civil, Environmental and Architectural Engineering, Korea University, Anam-Dong, Seongbuk-Gu, Seoul 136-713, South Korea

2-College of Pharmacy, Korea University, Sejong-ro 2511, Jochiwon-eup, Sejong, 339-700, South Korea

Construction of a pUCPLasR expression plasmid

LasR protein was ectopically expressed using a multicopy plasmid, pUCP18[1]. The sequence of the *lasR* gene region was amplified by PCR using Pfu polymerase (PfuUltra II Fusion HS DNA Polymerase, Agilent Technologies, Santa Clara, CA, USA) with the following oligonucleotides: 5’- ACCGAATTCCGGGATTCTCGGACTGCCGTAC (EcoRI restriction site is underlined) and 5’-CAGAAGCTTGCCGCTTCGGGATAAGCC (HindIII restriction site is underlined)(Supplementary Table 4). The amplified 951-bp fragment and plasmid pUCP18 were digested with EcoRI and HindIII endonuclease, gel purified, and ligated using T4 ligase (Promega, Madison, WI, USA). The ligated plasmid was transformed into *Escherichia coli* (XL10-Gold Ultracompetent cells, Agilent Technologies).

P. aeruginosa competent cells and transformation by electroporation

The preparation of *P. aeruginosa* competent cells and the transformation of *P. aeruginosa* using electroporation methods were based on a previous method[2]. 1.5 mL of overnight culture of *P. aeruginosa* cells in LB medium were harvested by centrifugation at room temperature for 3 min at 16,000 x g. The cell pellet was washed twice using 1 mL of 300 mM sucrose, and then the cell pellet was resuspended in 100 μL of 300 mM sucrose. For electroporation, purified plasmid DNA (pUCP18 and pUCPLasR) using the Qiagen plasmid prep kit (Qiagen, Chatsworth, CA, USA) was mixed with 100 μL of *P. aeruginosa* competent cells. The mixture was transferred to a 0.2 cm gap width electroporation cuvette (BIO-RAD, Hercules, CA, USA) and pulsed (25 μF, 200 Ω, 2.5 kV) using the Gene Pulser Xcell™ electroporation system (BIO-RAD). The electroporated cells (100 μL) were transferred to a 15-ml conical tube, 1 mL of fresh LB medium was added, and the mixture was shaken at 250 rpm at 37°C for 1 hr. The cells were spread on an LB plate with 100 μg/mL carbenicillin. Colonies were picked and grown in LB media. Plasmid DNA was isolated, treated with EcoRI and HindIII endonucleases, and separated using gel electrophoresis, to confirm insertion of the *lasR* gene into the plasmid.

Reference

1. Schweizer HP. Escherichia-Pseudomonas Shuttle Vectors Derived from Puc18 19. *Gene* **97**, 109-112 (1991).

2. Choi KH, Kumar A, Schweizer HP. A 10-min method for preparation of highly electrocompetent Pseudomonas aeruginosa cells: Application for DNA fragment transfer between chromosomes and plasmid transformation. *Journal of microbiological methods* **64**, 391-397 (2006).

Supplementary Fig. 1. (Kim et al)

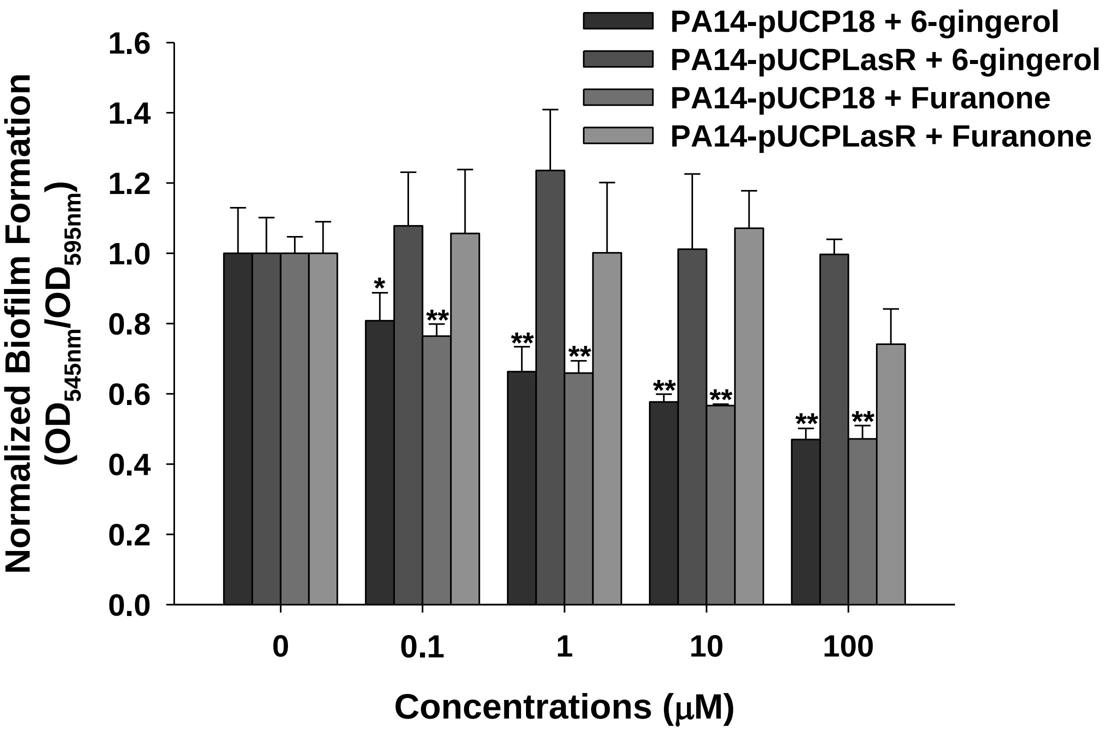

Supplementary figure 1. Quantification of PA14 biofilm formed in the wells of microtiter plates for PA14-pUCP18 and PA14-pUCPLasR with the addition of 6-gingerol (0 – 100 μM) and furanone C-30 (0 – 100 μM). Furanone C-30 was used as a positive control of an AHL analogue. The biofilm was quantified at 24 h of incubation by dividing OD at 545 nm by OD at 595 nm for cells stained with crystal violet. Error bars indicate the standard deviations of 10 measurements. *, $P<0.05$, and **, $P<0.005$ versus the control.

Supplymentary data 1. Activated and repressed genes by 6-gingerol based on a 1.5-fold cutoff in *P. aeruginosa* biofilm cells

PA gene	Gene	Description	Fold Change
PA0023	*qor*	quinone oxidoreductase	-1.6
PA0026	*plcB*	hypothetical protein	-1.8
PA0032		probable transcriptional regulator	1.5
PA0033		hypothetical protein	-1.5
PA0044	*exoT*	exoenzyme T	-3.4
PA0046		hypothetical protein	-2.4
PA0050		hypothetical protein	1.7
PA0053		hypothetical protein	1.5
PA0058		hypothetical protein	1.6
PA0059	*osmC*	osmotically inducible protein OsmC	-1.6
PA0066		conserved hypothetical protein	1.7
PA0079		hypothetical protein	-1.9
PA0097		hypothetical protein	1.5
PA0108	*coIII*	cytochrome c oxidase subunit III	-2.0
PA0139	*ahpC*	alkyl hydroperoxide reductase subunit C	-2.1
PA0140	*ahpF*	alkyl hydroperoxide reductase subunit F	-1.6
PA0165		hypothetical protein	-1.8
PA0183	*atsA*	arylsulfatase	1.8
PA0190		probable acid phosphatase	1.6
PA0200		hypothetical protein	-2.5
PA0201		hypothetical protein	1.8
PA0207		probable transcriptional regulator	2.7
PA0209		conserved hypothetical protein	-1.7
PA0211	*mdcD*	malonate decarboxylase beta subunit	1.5
PA0220		probable amino acid permease	-1.5
PA0222		hypothetical protein	1.5
PA0266	*gabT*	4-aminobutyrate aminotransferase	-1.5
PA0270		hypothetical protein	1.7
PA0284		hypothetical protein	3.1
PA0299	*spuC*	probable aminotransferase	-1.7
PA0318		conserved hypothetical protein	-1.6
PA0342	*thyA*	thymidylate synthase	-1.7
PA0375	*ftsX*	cell division protein FtsX	-1.8
PA0376	*rpoH*	sigma factor RpoH	2.3
PA0390	*metX*	homoserine O-acetyltransferase	1.7
PA0404		conserved hypothetical protein	-1.5
PA0406		hypothetical protein	-1.7
PA0409	*pilH*	twitching motility protein PilH	-1.7
PA0413	*chpA*	still frameshift probable component of chemotactic signal transduction system	-1.9
PA0415	*chpC*	probable chemotaxis protein	-1.8
PA0424	*mexR*	multidrug resistance operon repressor MexR	-1.6
PA0425	*mexA*	RND multidrug efflux membrane fusion protein MexA precursor	-1.6
PA0427	*oprM*	outer membrane protein OprM precursor	-1.6
PA0431		hypothetical protein	-2.3
PA0432	*sahH*	S-adenosyl-L-homocysteine hydrolase	-2.2
PA0434		hypothetical protein	1.7
PA0457		hypothetical protein	-1.5
PA0459		probable ClpA/B protease ATP binding subunit	-2.3
PA0480		probable hydrolase	2.1
PA0491		probable transcriptional regulator	1.8
PA0499		probable pili assembly chaperone	1.9
PA0541		hypothetical protein	-1.6
PA0546	*metK*	methionine adenosyltransferase	-1.8
PA0566		hypothetical protein	-3.3
PA0576	*rpoD*	sigma factor RpoD	-1.7
PA0577	*dnaG*	DNA primase	-1.7
PA0578		conserved hypothetical protein	-1.8
PA0579	*rpsU*	30S ribosomal protein S21	-1.5
PA0599		hypothetical protein	1.8
PA0608		probable phosphoglycolate phosphatase	-1.7
PA0610	*prtN*	transcriptional regulator PrtN	2.7
PA0612		hypothetical protein	1.9
PA0617		probable bacteriophage protein	2.8
PA0620		probable bacteriophage protein	1.7
PA0621		conserved hypothetical protein	1.6

PA0622		probable bacteriophage protein	2.2
PA0623		probable bacteriophage protein	2.4
PA0624		hypothetical protein	2.1
PA0625		hypothetical protein	1.7
PA0629		conserved hypothetical protein	2.7
PA0630		hypothetical protein	1.5
PA0631		hypothetical protein	2.2
PA0632		hypothetical protein	1.7
PA0633		hypothetical protein	4.5
PA0635		hypothetical protein	2.9
PA0636		hypothetical protein	2.0
PA0637		conserved hypothetical protein	1.6
PA0639		conserved hypothetical protein	2.9
PA0641		probable bacteriophage protein	1.6
PA0643		hypothetical protein	1.9
PA0646		hypothetical protein	1.8
PA0648		hypothetical protein	1.5
PA0652	*vfr*	transcriptional regulator Vfr	-3.4
PA0654	*speD*	S-adenosylmethionine decarboxylase proenzyme	-1.6
PA0665		conserved hypothetical protein	1.7
PA0667		conserved hypothetical protein	-1.6
PA0672	*hemO*	hypothetical protein	2.4
PA0689		hypothetical protein	2.0
PA0695		hypothetical protein	1.6
PA0709		hypothetical protein	1.7
PA0713		hypothetical protein	2.2
PA0720		helix destabilizing protein of bacteriophage Pf1	1.5
PA0723	*coaB*	coat protein B of bacteriophage Pf1)	1.5
PA0762	*algU*	sigma factor AlgU	2.2
PA0763	*mucA*	anti-sigma factor MucA	1.7
PA0767	*lepA*	GTP-binding protein LepA	-1.7
PA0779		probable ATP-dependent protease	1.5
PA0782	*putA*	proline dehydrogenase PutA	-1.6
PA0794		probable aconitate hydratase	2.6
PA0797		probable transcriptional regulator	1.5
PA0801		hypothetical protein	1.7
PA0807		conserved hypothetical protein	5.7
PA0814		conserved hypothetical protein	1.6
PA0816		probable transcriptional regulator	1.7
PA0820		hypothetical protein	1.6
PA0821		hypothetical protein	-1.6
PA0825		hypothetical protein	-2.3
PA0833		hypothetical protein	2.1
PA0837	*slyD*	peptidyl-prolyl cis-trans isomerase SlyD	-1.9
PA0861		hypothetical protein	1.7
PA0862		hypothetical protein	-1.7
PA0864		probable transcriptional regulator	1.6
PA0865	*hpd*	4-hydroxyphenylpyruvate dioxygenase	1.7
PA0894		hypothetical protein	1.6
PA0905	*rsmA*	carbon storage regulator	2.6
PA0907		hypothetical protein	1.8
PA0915		conserved hypothetical protein	-1.6
PA0916		conserved hypothetical protein	-1.7
PA0937		conserved hypothetical protein	-1.7
PA0942		probable transcriptional regulator	1.7
PA0948		hypothetical protein	-1.5
PA0949	*wrbA*	Trp repressor binding protein WrbA	-1.7
PA0962		probable dna-binding stress protein	-1.6
PA0966	*ruvA*	Holliday junction DNA helicase RuvA	-1.5
PA0968		conserved hypothetical protein	1.5
PA0973	*oprL*	outer membrane protein OprL precursor	-1.5
PA0979		conserved hypothetical protein	-1.6
PA0983		conserved hypothetical protein	1.7
PA0984		colicin immunity protein	1.6
PA0997	*pqsB*	hypothetical protein	-3.0
PA0998	*pqsC*	hypothetical protein	-3.4
PA0999	*pqsD*	3-oxoacyl-[acyl-carrier-protein] synthase III	-2.3
PA1000	*pqsE*	hypothetical protein	-4.7

PA1001	*phnA*	anthranilate synthase component I	-3.5
PA1002	*phnB*	anthranilate synthase component II	-2.3
PA1009		hypothetical protein	-2.1
PA1025		probable porin	2.0
PA1048		probable outer membrane protein	-1.7
PA1053		conserved hypothetical protein	-2.7
PA1069		hypothetical protein	-1.6
PA1077	*flgB*	flagellar basal-body rod protein FlgB	-2.3
PA1078	*flgC*	flagellar basal-body rod protein FlgC	-2.4
PA1080	*flgE*	flagellar hook protein FlgE	-4.6
PA1081	*flgF*	flagellar basal-body rod protein FlgF	-3.8
PA1082	*flgG*	flagellar basal-body rod protein FlgG	-2.3
PA1084	*flgI*	flagellar P-ring protein precursor FlgI	-1.8
PA1085	*flgJ*	flagellar protein FlgJ	-2.9
PA1087	*flgL*	flagellar hook-associated protein type 3 FlgL	-2.6
PA1089		conserved hypothetical protein	-2.6
PA1091		hypothetical protein	-3.3
PA1092	*fliC*	flagellin type B	2.7
PA1093		hypothetical protein	1.8
PA1095		hypothetical protein	1.9
PA1099	*fleR*	two-component response regulator	-1.7
PA1102	*fliG*	flagellar motor switch protein FliG	-2.7
PA1103		probable flagellar assembly protein	-4.2
PA1118		hypothetical protein	1.8
PA1133		hypothetical protein	1.9
PA1142		probable transcriptional regulator	2.1
PA1143		hypothetical protein	2.1
PA1152		hypothetical protein	1.6
PA1153		hypothetical protein	1.5
PA1166		hypothetical protein	2.0
PA1178	*oprH*	outer membrane protein H1 precursor	-2.0
PA1187		probable acyl-CoA dehydrogenase	-1.5
PA1190		conserved hypothetical protein	1.9
PA1224		probable NAD(P)H dehydrogenase	2.0
PA1230		hypothetical protein	-1.7
PA1255		hypothetical protein	1.8
PA1264		probable transcriptional regulator	1.6
PA1275	*cobD*	cobalamin biosynthetic protein CobD	2.0
PA1278	*cobP*	cobinamide kinase	-1.5
PA1287		probable glutathione peroxidase	1.6
PA1302		probable heme utilization protein precursor	1.7
PA1303		probable signal peptidase	1.6
PA1325		conserved hypothetical protein	1.5
PA1331		conserved hypothetical protein	1.6
PA1332		hypothetical protein	1.5
PA1342		probable binding protein component of ABC transporter	-1.8
PA1358		hypothetical protein	1.5
PA1367		hypothetical protein	2.0
PA1387		hypothetical protein	1.8
PA1392		hypothetical protein	1.7
PA1429		probable cation-transporting P-type ATPase	1.5
PA1431	*rsaL*	regulatory protein RsaL	1.5
PA1433		conserved hypothetical protein	1.6
PA1453	*flhF*	flagellar biosynthesis protein FlhF	1.5
PA1458		probable two-component sensor	-1.7
PA1470		probable short-chain dehydrogenase	1.5
PA1477	*ccmC*	heme exporter protein CcmC	1.6
PA1479	*ccmE*	cytochrome C-type biogenesis protein CcmE	-1.6
PA1486		hypothetical protein	1.8
PA1489		hypothetical protein	1.9
PA1504		probable transcriptional regulator	-1.9
PA1506		hypothetical protein	-1.5
PA1515	*alc*	allantoicase	1.6
PA1529	*lig*	DNA ligase	-1.7
PA1540		conserved hypothetical protein	-1.6
PA1553		probable cytochrome c oxidase subunit	-1.7
PA1555		probable cytochrome c	-1.8
PA1567		conserved hypothetical protein	1.5

PA1568		conserved hypothetical protein	1.7
PA1573		conserved hypothetical protein	-1.5
PA1581	*sdhC*	succinate dehydrogenase (C subunit)	-2.2
PA1582	*sdhD*	succinate dehydrogenase (D subunit)	-1.8
PA1584	*sdhB*	succinate dehydrogenase (B subunit)	-1.7
PA1586	*sucB*	dihydrolipoamide succinyltransferase (E2 subunit)	-1.6
PA1593		hypothetical protein	-1.9
PA1602		probable oxidoreductase	-1.6
PA1608		probable chemotaxis transducer	-1.6
PA1614	*gpsA*	glycerol-3-phosphate dehydrogenase biosynthetic	1.6
PA1638		conserved hypothetical protein	1.6
PA1692		probable translocation protein in type III secretion	-3.1
PA1694	*pscQ*	translocation protein in type III secretion	-1.8
PA1695	*pscP*	translocation protein in type III secretion	-1.8
PA1698	*popN*	outer membrane protein PopN	-2.0
PA1699		conserved hypothetical protein in type III secretion	-2.2
PA1700		conserved hypothetical protein in type III secretion	-3.2
PA1701		conserved hypothetical protein in type III secretion	-2.8
PA1702		conserved hypothetical protein in type III secretion	-3.0
PA1703	*pcrD*	type III secretory apparatus protein PcrD	-2.2
PA1704	*pcrR*	transcriptional regulator protein PcrR	-1.7
PA1705	*pcrG*	regulator in type III secretion	-2.2
PA1706	*pcrV*	type III secretion protein PcrV	-3.3
PA1708	*popB*	translocator protein PopB	-2.9
PA1709	*popD*	translocator protein PopD	-1.7
PA1710	*exsC*	exoenzyme S synthesis protein C precursor	-3.0
PA1714		hypothetical protein	-2.6
PA1715	*pscB*	type III export apparatus protein	-2.4
PA1716	*pscC*	type III secretion protein PscC	-2.6
PA1717	*pscD*	type III export protein PscD	-3.8
PA1719	*pscF*	type III export protein PscF	-1.8
PA1720	*pscG*	type III export protein PscG	-2.9
PA1721	*pscH*	type III export protein PscH	-2.9
PA1722	*pscI*	type III export protein PscI	-1.8
PA1723	*pscJ*	type III export protein PscJ	-3.0
PA1724	*pscK*	type III export protein PscK	-2.5
PA1735		hypothetical protein	1.7
PA1754	*cysB*	transcriptional regulator CysB	-1.6
PA1763		hypothetical protein	-1.6
PA1767		hypothetical protein	-2.2
PA1772		probable methyltransferase	-2.1
PA1777	*oprF*	outer membrane protein OprF precursor	-1.8
PA1813		probable hydroxyacylglutathione hydrolase	-1.9
PA1814		hypothetical protein	1.9
PA1832		probable protease	1.5
PA1834		hypothetical protein	-1.8
PA1852		hypothetical protein	1.5
PA1860		hypothetical protein	1.6
PA1868	*xqhA*	secretion protein XqhA	1.5
PA1889		hypothetical protein	1.7
PA1901	*phzC1 /// phzC2*	phenazine biosynthesis protein PhzC	-36.9
PA1902	*phzD1 /// phzD2*	phenazine biosynthesis protein PhzD	-2.0
PA1903	*phzE1 /// phzE2*	phenazine biosynthesis protein PhzE	-13.7
PA1904	*phzF1 /// phzF2*	probable phenazine biosynthesis protein	-2.1
PA1905	*phzG2*	probable pyridoxamine 5'-phosphate oxidase	-9.6
PA1907		hypothetical protein	1.7
PA1916		probable amino acid permease	1.6
PA1923		hypothetical protein	1.5
PA1932		probable hydroxylase molybdopterin-containing subunit	1.6
PA1942		hypothetical protein	-4.8
PA1952		hypothetical protein	1.9
PA1953		hypothetical protein	1.6
PA1977		hypothetical protein	2.9
PA1991		probable iron-containing alcohol dehydrogenase	1.5
PA2009	*hmgA*	homogentisate 12-dioxygenase	-1.8
PA2025	*gor*	glutathione reductase	1.8
PA2029		hypothetical protein	1.8
PA2031		hypothetical protein	-1.7

PA2046		hypothetical protein	2.1
PA2048		hypothetical protein	1.6
PA2062		probable pyridoxal-phosphate dependent enzyme	1.7
PA2074		hypothetical protein	2.1
PA2078		hypothetical protein	1.6
PA2091		hypothetical protein	1.6
PA2096		probable transcriptional regulator	1.7
PA2098		probable esterase/deacetylase	1.5
PA2108		probable decarboxylase	1.5
PA2119		alcohol dehydrogenase (Zn-dependent)	1.6
PA2127		conserved hypothetical protein	-1.7
PA2167		hypothetical protein	1.5
PA2172		hypothetical protein	1.5
PA2173		hypothetical protein	2.0
PA2185	*katN*	hypothetical protein	1.6
PA2186		hypothetical protein	1.7
PA2191	*exoY*	adenylate cyclase ExoY	-1.7
PA2198		hypothetical protein	1.5
PA2216		conserved hypothetical protein	1.6
PA2233	*pslC*	probable glycosyl transferase	1.5
PA2245	*pslO*	hypothetical protein	1.7
PA2274		hypothetical protein	-2.3
PA2288		hypothetical protein	3.6
PA2302		probable non-ribosomal peptide synthetase	1.8
PA2308		probable ATP-binding component of ABC transporter	1.6
PA2320	*gntR*	transcriptional regulator GntR	-1.7
PA2331		hypothetical protein	2.5
PA2335		probable TonB-dependent receptor	1.6
PA2336		hypothetical protein	1.5
PA2339		probable binding-protein-dependent maltose/mannitol transport protein	-1.5
PA2340		probable binding-protein-dependent maltose/mannitol transport protein	-1.7
PA2356	*msuD*	methanesulfonate sulfonatase MsuD	1.5
PA2361		hypothetical protein	1.6
PA2381		hypothetical protein	-2.8
PA2383		probable transcriptional regulator	-1.8
PA2384		hypothetical protein	-2.7
PA2385	*pvdQ*	probable acylase	-1.7
PA2386	*pvdA*	L-ornithine N5-oxygenase	-1.9
PA2387		probable sigma-70 factor ECF subfamily	1.6
PA2421		hypothetical protein	2.0
PA2433		hypothetical protein	1.9
PA2437		hypothetical protein	1.6
PA2458		hypothetical protein	-1.8
PA2467		probable transmembrane sensor	1.6
PA2559		hypothetical protein	-1.7
PA2561		probable chemotaxis transducer	1.6
PA2577		probable transcriptional regulator	-1.7
PA2594		conserved hypothetical protein	2.0
PA2599		conserved hypothetical protein	1.6
PA2601		probable transcriptional regulator	2.2
PA2602		hypothetical protein	1.9
PA2604		conserved hypothetical protein	2.2
PA2622	*cspD*	cold-shock protein CspD	-1.9
PA2624	*idh*	isocitrate dehydrogenase	-1.6
PA2626	*trmU*	tRNA methyltransferase	-3.2
PA2630		conserved hypothetical protein	-1.5
PA2641	*nuoF*	NADH dehydrogenase I chain F	-1.5
PA2643	*nuoH*	NADH dehydrogenase I chain H	-1.5
PA2648	*nuoM*	NADH dehydrogenase I chain M	-1.6
PA2651		conserved hypothetical protein	2.0
PA2655		hypothetical protein	2.0
PA2671		hypothetical protein	1.8
PA2706		hypothetical protein	1.6
PA2719		hypothetical protein	1.6
PA2721		hypothetical protein	2.0
PA2732		hypothetical protein	1.8
PA2733		conserved hypothetical protein	2.0
PA2755	*eco*	ecotin precursor	-1.7

PA2758		probable transcriptional regulator	1.6
PA2773		hypothetical protein	1.5
PA2794		hypothetical protein	1.6
PA2818		hypothetical protein	1.7
PA2826		probable glutathione peroxidase	2.2
PA2828		probable aminotransferase	1.7
PA2831		conserved hypothetical protein	1.8
PA2832	*tpm*	thiopurine methyltransferase	1.5
PA2845		hypothetical protein	1.9
PA2862	*lipA*	lactonizing lipase precursor	-1.5
PA2868		hypothetical protein	-1.5
PA2870		hypothetical protein	-1.6
PA2872		hypothetical protein	-2.1
PA2877		probable transcriptional regulator	1.6
PA2883		hypothetical protein	-1.8
PA2886		hypothetical protein	1.7
PA2887		probable short-chain dehydrogenase	2.0
PA2890		probable enoyl-CoA hydratase/isomerase	1.7
PA2891		probable biotin carboxylase/biotin carboxyl carrier protein	1.9
PA2898		hypothetical protein	1.6
PA2912		probable ATP-binding component of ABC transporter	2.0
PA2931		probable transcriptional regulator	2.5
PA2932	*morB*	morphinone reductase	2.1
PA2951	*etfA*	electron transfer flavoprotein alpha-subunit	-2.3
PA2952	*etfB*	electron transfer flavoprotein beta-subunit	-2.5
PA2962	*tmk*	thymidylate kinase	-1.9
PA2971		conserved hypothetical protein	1.8
PA2983		probable tolQ-type transport protein	-1.9
PA2996	*nqrD*	Na+-translocating NADH:uniquinone oxidoreductase subunit Nqr4	-1.6
PA3013	*foaB*	fatty-acid oxidation complex beta-subunit	-3.3
PA3031		hypothetical protein	1.7
PA3043		conserved hypothetical protein	1.6
PA3061	*pelD*	hypothetical protein	-1.5
PA3065		hypothetical protein	1.6
PA3068	*gdhB*	conserved hypothetical protein	-1.5
PA3096	*xcpY*	general secretion pathway protein L	-1.5
PA3104	*xcpP*	secretion protein XcpP	-1.8
PA3105	*xcpQ*	general secretion pathway protein D	-1.5
PA3109		hypothetical protein	1.8
PA3113	*trpF*	N-(5'phosphoribosyl)anthranilate (PRA) isomerase	-1.8
PA3126	*ibpA*	heat-shock protein IbpA	3.1
PA3130		hypothetical protein	1.6
PA3139		probable amino acid aminotransferase	-1.6
PA3140		hypothetical protein	1.6
PA3157		probable acetyltransferase	1.6
PA3167	*serC*	3-phosphoserine aminotransferase	-1.7
PA3177		hypothetical protein	-1.9
PA3181		2-keto-3-deoxy-6-phosphogluconate aldolase	-1.7
PA3182	*pgl*	conserved hypothetical protein	-3.9
PA3188		probable permease of ABC sugar transporter	-2.0
PA3190		probable binding protein component of ABC sugar transporter	-2.0
PA3194	*edd*	phosphogluconate dehydratase	-2.3
PA3195	*gapA*	glyceraldehyde 3-phosphate dehydrogenase	-1.6
PA3205		hypothetical protein	-1.7
PA3213		hypothetical protein	1.8
PA3234		probable sodium:solute symporter	-1.6
PA3246	*rluA*	pseudouridine synthase RluA	-1.8
PA3258		hypothetical protein	-1.8
PA3272		probable ATP-dependent DNA helicase	1.5
PA3295		probable HIT family protein	1.6
PA3314		probable ATP-binding component of ABC transporter	2.1
PA3315		probable permease of ABC transporter	1.6
PA3317		hypothetical protein	1.6
PA3318		hypothetical protein	1.6
PA3328		probable FAD-dependent monooxygenase	1.6
PA3332		conserved hypothetical protein	-1.7
PA3349		probable chemotaxis protein	1.9
PA3373		conserved hypothetical protein	1.5

PA3376		probable ATP-binding component of ABC transporter	2.2
PA3382	*phnE*	phosphonate transport protein PhnE	1.6
PA3385		hypothetical protein	2.1
PA3409		probable transmembrane sensor	1.6
PA3414		hypothetical protein	1.6
PA3415		probable dihydrolipoamide acetyltransferase	1.5
PA3444		conserved hypothetical protein	2.3
PA3446		conserved hypothetical protein	1.7
PA3450		probable antioxidant protein	2.1
PA3458		probable transcriptional regulator	-1.5
PA3463		conserved hypothetical protein	-1.6
PA3477	*rhlR*	transcriptional regulator RhlR	-1.6
PA3478	*rhlB*	rhamnosyltransferase chain B	-1.5
PA3479	*rhlA*	rhamnosyltransferase chain A	-3.6
PA3496		hypothetical protein	1.7
PA3499		hypothetical protein	1.7
PA3503		hypothetical protein	1.6
PA3507		probable short-chain dehydrogenase	1.6
PA3511		probable short-chain dehydrogenase	1.9
PA3526		probable outer membrane protein	1.7
PA3530		conserved hypothetical protein	9.7
PA3533		conserved hypothetical protein	1.8
PA3536		hypothetical protein	1.9
PA3555		conserved hypothetical protein	1.6
PA3560	*fruA*	phosphotransferase system fructose-specific IIBC component	2.0
PA3561	*fruK*	1-phosphofructokinase	1.7
PA3568		probable acetyl-coa synthetase	2.3
PA3570	*mmsA*	methylmalonate-semialdehyde dehydrogenase	-1.5
PA3577		hypothetical protein	-1.9
PA3584	*glpD*	glycerol-3-phosphate dehydrogenase	-1.5
PA3590		probable hydroxyacyl-CoA dehydrogenase	1.7
PA3600		conserved hypothetical protein	2.0
PA3601		conserved hypothetical protein	1.9
PA3607	*potA*	polyamine transport protein PotA	-1.8
PA3616		conserved hypothetical protein	1.5
PA3617	*recA*	RecA protein	2.7
PA3618		conserved hypothetical protein	-1.9
PA3650	*dxr*	1-deoxy-d-xylulose 5-phosphate reductoisomerase	-1.5
PA3654	*pyrH*	uridylate kinase	-2.8
PA3692		probable outer membrane protein	-1.8
PA3704	*wspE*	probable chemotaxis sensor/effector fusion protein	1.6
PA3719		hypothetical protein	1.8
PA3724	*lasB*	elastase LasB	-1.7
PA3744	*rimM*	16S rRNA processing protein	-1.6
PA3745	*rpsP*	30S ribosomal protein S16	-3.0
PA3747		conserved hypothetical protein	-1.8
PA3758		probable N-acetylglucosamine-6-phosphate deacetylase	1.6
PA3763	*purL*	phosphoribosylformylglycinamidine synthase	-2.0
PA3769	*guaA*	GMP synthase	1.7
PA3775		hypothetical protein	1.5
PA3784		hypothetical protein	-1.5
PA3791		hypothetical protein	-1.9
PA3792	*leuA*	2-isopropylmalate synthase	-2.1
PA3801		conserved hypothetical protein	-1.8
PA3804		hypothetical protein	-1.8
PA3815		conserved hypothetical protein	2.8
PA3820	*secF*	secretion protein SecF	-2.1
PA3827		conserved hypothetical protein	-1.7
PA3828		conserved hypothetical protein	-1.7
PA3842		probable chaperone	-1.6
PA3880		conserved hypothetical protein	-1.7
PA3887	*nhaP*	Na+/H+ antiporter NhaP	-1.5
PA3897		hypothetical protein	1.5
PA3899		probable sigma-70 factor ECF subfamily	1.6
PA3900		probable transmembrane sensor	2.3
PA3909		hypothetical protein	1.6
PA3911		conserved hypothetical protein	-1.6
PA3926		probable MFS transporter	2.9

PA3927		probable transcriptional regulator	1.7
PA3930	*cioA*	cyanide insensitive terminal oxidase	-2.6
PA3961		probable ATP-dependent helicase	-1.6
PA3964		hypothetical protein	1.6
PA3982		conserved hypothetical protein	-1.5
PA4003	*pbpA*	penicillin-binding protein 2	-1.7
PA4008		probable hydrolase	1.7
PA4014		hypothetical protein	-1.6
PA4015		conserved hypothetical protein	-1.7
PA4019		probable aromatic acid decarboxylase	-2.0
PA4028		hypothetical protein	1.5
PA4042	*xseB*	exodeoxyribonuclease VII small subunit	-2.6
PA4045		conserved hypothetical protein	-1.6
PA4049		hypothetical protein	-2.2
PA4055	*ribC*	riboflavin synthase alpha chain	1.5
PA4059		hypothetical protein	-2.4
PA4070		probable transcriptional regulator	1.9
PA4075		hypothetical protein	1.6
PA4096		probable MFS transporter	1.6
PA4102		probable two-component sensor	1.8
PA4130		probable sulfite or nitrite reductase	-2.7
PA4131		probable iron-sulfur protein	-5.7
PA4132		conserved hypothetical protein	-2.2
PA4134		hypothetical protein	-5.2
PA4139		hypothetical protein	-1.6
PA4141		hypothetical protein	-10.6
PA4142		probable secretion protein	-1.7
PA4155		hypothetical protein	-1.6
PA4156		probable TonB-dependent receptor	-1.6
PA4159	*fepB*	ferrienterobactin-binding periplasmic protein precursor FepB	2.0
PA4163		hypothetical protein	-1.7
PA4165		probable transcriptional regulator	1.7
PA4178		hypothetical protein	2.0
PA4185		probable transcriptional regulator	-1.5
PA4195		probable binding protein component of ABC transporter	1.6
PA4207	*mexI*	probable RND efflux transporter	-2.1
PA4211	*phzB1 /// phzB2*	probable phenazine biosynthesis protein	-1.7
PA4220		hypothetical protein	-1.5
PA4221	*fptA*	Fe(III)-pyochelin receptor precursor	-1.6
PA4223		probable ATP-binding component of ABC transporter	-1.5
PA4224	*pchG*	hypothetical protein	-2.5
PA4229	*pchC*	pyochelin biosynthetic protein PchC	-1.6
PA4238	*rpoA*	DNA-directed RNA polymerase alpha chain	-2.3
PA4240	*rpsK*	30S ribosomal protein S11	-2.3
PA4242	*rpmJ*	50S ribosomal protein L36	-2.1
PA4248	*rplF*	50S ribosomal protein L6	-1.5
PA4249	*rpsH*	30S ribosomal protein S8	-1.5
PA4250	*rpsN*	30S ribosomal protein S14	-1.7
PA4251	*rplE*	50S ribosomal protein L5	-3.0
PA4254	*rpsQ*	30S ribosomal protein S17	-1.5
PA4256	*rplP*	50S ribosomal protein L16	-1.6
PA4259	*rpsS*	30S ribosomal protein S19	-2.1
PA4260	*rplB*	50S ribosomal protein L2	-2.0
PA4261	*rplW*	50S ribosomal protein L23	-1.6
PA4262	*rplD*	50S ribosomal protein L4	-2.1
PA4268	*rpsL*	30S ribosomal protein S12	1.7
PA4269	*rpoC*	DNA-directed RNA polymerase beta* chain	-4.3
PA4272	*rplJ*	50S ribosomal protein L10	-1.8
PA4273	*rplA*	50S ribosomal protein L1	-2.1
PA4274	*rplK*	50S ribosomal protein L11	-3.6
PA4279		hypothetical protein	-1.8
PA4284	*recB*	exodeoxyribonuclease V beta chain	1.6
PA4288		probable transcriptional regulator	1.8
PA4326		hypothetical protein	1.5
PA4329	*pykA*	pyruvate kinase II	-1.6
PA4345		hypothetical protein	1.6
PA4352		conserved hypothetical protein	-3.1
PA4355		probable MFS transporter	1.8

PA4359		conserved hypothetical protein	2.5
PA4365		probable transporter	1.6
PA4369		hypothetical protein	1.5
PA4374		probable RND efflux membrane fusion protein precursor	1.5
PA4377		hypothetical protein	-1.5
PA4379		conserved hypothetical protein	-1.5
PA4385	*groEL*	GroEL protein	2.0
PA4386	*groES*	GroES protein	3.5
PA4394		conserved hypothetical protein	-1.6
PA4395		conserved hypothetical protein	-2.6
PA4402	*argJ*	glutamate N-acetyltransferase	1.5
PA4403	*secA*	secretion protein SecA	-1.9
PA4406	*lpxC*	UDP-3-O-acyl-N-acetylglucosamine deacetylase	1.5
PA4424		conserved hypothetical protein	-2.0
PA4425		probable phosphoheptose isomerase	-1.9
PA4426		conserved hypothetical protein	-2.0
PA4437		hypothetical protein	-1.6
PA4441		hypothetical protein	-2.0
PA4451		conserved hypothetical protein	-3.1
PA4454		conserved hypothetical protein	-1.9
PA4457		conserved hypothetical protein	1.6
PA4459		conserved hypothetical protein	-1.5
PA4464	*ptsN*	nitrogen regulatory IIA protein	-1.8
PA4467		hypothetical protein	2.1
PA4471		hypothetical protein	2.2
PA4473		hypothetical protein	-1.6
PA4481	*mreB*	rod shape-determining protein MreB	-1.6
PA4482	*gatC*	Glu-tRNA(Gln) amidotransferase subunit C	-1.7
PA4490		conserved hypothetical protein	-1.6
PA4492		conserved hypothetical protein	-1.6
PA4500		probable binding protein component of ABC transporter	-1.6
PA4504		probable permease of ABC transporter	2.3
PA4507		hypothetical protein	-1.5
PA4511		conserved hypothetical protein	-1.7
PA4523		hypothetical protein	1.7
PA4536		hypothetical protein	-1.8
PA4537		hypothetical protein	-3.6
PA4542	*clpB*	ClpB protein	1.7
PA4547	*pilR*	two-component response regulator PilR	-1.7
PA4558		probable peptidyl-prolyl cis-trans isomerase FkbP-type	-2.4
PA4563	*rpsT*	30S ribosomal protein S20	-2.2
PA4568	*rplU*	50S ribosomal protein L21	-1.8
PA4570		hypothetical protein	2.6
PA4573		hypothetical protein	-1.7
PA4578		hypothetical protein	-2.2
PA4592		hypothetical protein	1.5
PA4594		probable ATP-binding component of ABC transporter	2.0
PA4599	*mexC*	RND multidrug efflux membrane fusion protein MexC precursor	1.6
PA4603		hypothetical protein	1.7
PA4644		hypothetical protein	1.6
PA4646	*upp*	uracil phosphoribosyltransferase	-1.6
PA4657		hypothetical protein	1.6
PA4665	*prfA*	peptide chain release factor 1	-2.0
PA4667		hypothetical protein	-1.6
PA4670	*prs*	ribose-phosphate pyrophosphokinase	-1.6
PA4671		probable ribosomal protein L25	-2.1
PA4697		hypothetical protein	-2.4
PA4698		hypothetical protein	-1.7
PA4700	*mrcB*	penicillin-binding protein 1B	-2.1
PA4729	*panB*	3-methyl-2-oxobutanoate hydroxymethyltransferase	-2.0
PA4731	*panD*	aspartate 1-decarboxylase precursor	-1.5
PA4742	*truB*	tRNA pseudouridine 55 synthase	-1.6
PA4744	*infB*	translation initiation factor IF-2	-2.5
PA4745	*nusA*	N utilization substance protein A	-2.5
PA4749	*glmM*	phosphoglucosamine mutase	-1.7
PA4750	*folP*	dihydropteroate synthase	1.6
PA4751	*ftsH*	cell division protein FtsH	-2.0
PA4754		hypothetical protein	-1.8

PA4755	*greA*	transcription elongation factor GreA	-3.1
PA4756	*carB*	carbamoylphosphate synthetase large subunit	-1.6
PA4766		conserved hypothetical protein	1.6
PA4781		probable two-component response regulator	2.0
PA4784		probable transcriptional regulator	-1.9
PA4793		hypothetical protein	-1.7
PA4795		hypothetical protein	1.7
PA4804		probable amino acid permease	1.5
PA4809	*fdhE*	FdhE protein	1.6
PA4818		conserved hypothetical protein	1.8
PA4840		conserved hypothetical protein	1.6
PA4856	*rtsM*	probable sensor/response regulator hybrid	-1.9
PA4871		hypothetical protein	1.5
PA4883		hypothetical protein	1.5
PA4899		probable aldehyde dehydrogenase	1.6
PA4917		hypothetical protein	-1.7
PA4919	*pncB1*	nicotinate phosphoribosyltransferase	1.7
PA4921		hypothetical protein	-1.6
PA4926		conserved hypothetical protein	1.5
PA4932	*rplI*	50S ribosomal protein L9	-1.6
PA4933		hypothetical protein	-1.6
PA4938	*purA*	adenylosuccinate synthetase	-1.9
PA4939		conserved hypothetical protein	-1.9
PA4940		conserved hypothetical protein	-2.0
PA4947	*amiB*	N-acetylmuramoyl-L-alanine amidase	-1.7
PA4961		hypothetical protein	-1.5
PA5011	*waaC*	heptosyltransferase I	-1.5
PA5015	*aceE*	pyruvate dehydrogenase	-2.6
PA5016	*aceF*	dihydrolipoamide acetyltransferase	-1.8
PA5024		conserved hypothetical protein	-1.5
PA5027		hypothetical protein	-1.8
PA5045	*ponA*	penicillin-binding protein 1A	-2.3
PA5049	*rpmE*	50S ribosomal protein L31	1.8
PA5051	*argS*	arginyl-tRNA synthetase	1.7
PA5054	*hslU*	heat shock protein HslU	-1.5
PA5067	*hisE*	phosphoribosyl-ATP pyrophosphohydrolase	-2.2
PA5068	*tatA*	translocation protein TatA	-1.6
PA5091	*hutG*	N-formylglutamate amidohydrolase	-1.8
PA5094		probable ATP-binding component of ABC transporter	-1.7
PA5099		probable transporter	1.6
PA5108		hypothetical protein	1.6
PA5112	*estA*	esterase EstA	1.5
PA5117	*typA*	regulatory protein TypA	-1.6
PA5124	*ntrB*	two-component sensor NtrB	-1.5
PA5148		conserved hypothetical protein	-1.8
PA5157		probable transcriptional regulator	-1.6
PA5164	*rmlC*	dTDP-4-dehydrorhamnose 35-epimerase	-1.6
PA5195		probable heat shock protein	1.7
PA5215	*gcvT1*	glycine-cleavage system protein T1	1.5
PA5239	*rho*	transcription termination factor Rho	-1.6
PA5244		conserved hypothetical protein	-1.7
PA5253	*algP*	alginate regulatory protein AlgP	-1.6
PA5254		probable peptidyl-prolyl cis-trans isomerase FkbP-type	-1.6
PA5263	*argH*	argininosuccinate lyase	-2.3
PA5273		hypothetical protein	1.6
PA5279		conserved hypothetical protein	-2.0
PA5282		probable MFS transporter	1.9
PA5288	*glnK*	nitrogen regulatory protein P-II 2	-2.3
PA5323	*argB*	acetylglutamate kinase	-1.5
PA5325		hypothetical protein	-1.6
PA5345	*recG*	ATP-dependent DNA helicase RecG	-1.6
PA5347		hypothetical protein	1.9
PA5350	*rubA2*	rubredoxin	1.5
PA5351	*rubA1*	rubredoxin	-1.5
PA5374	*betI*	transcriptional regulator BetI	1.5
PA5379	*sdaB*	L-serine dehydratase	1.6
PA5414		hypothetical protein	-2.0
PA5416	*soxB*	sarcosine oxidase beta subunit	2.3

PA5446		hypothetical protein	-2.9
PA5453	*gmd*	GDP-mannose 46-dehydratase	-1.5
PA5458		hypothetical protein	-1.7
PA5466		hypothetical protein	1.8
PA5473		conserved hypothetical protein	-1.6
PA5475		hypothetical protein	-1.6
PA5490	*cc4*	cytochrome c4 precursor	-1.9
PA5512		probable two-component sensor	-1.8
PA5526		hypothetical protein	2.0
PA5537		hypothetical protein	1.7
PA5544		conserved hypothetical protein	1.7
PA5545		conserved hypothetical protein	1.7
PA5547		conserved hypothetical protein	-1.6
PA5559	*atpE*	atp synthase C chain	-1.6
PA5562	*spoOJ*	chromosome partitioning protein Spo0J	-2.2
PA5564	*gidB*	glucose inhibited division protein B	-1.7
PA5568		conserved hypothetical protein	-2.3
PA5569	*rnpA*	ribonuclease P protein component	-1.7

Gene numbers are annotated for *P. aeruginosa* PAO1.

Supplementary data 2. Expression of QS associated genes when treated with 6-gingerol compared to previous studies

PA gene	Gene	Description	Fold change			
			Henzter et al	Schuster et al	Wagner et al	This study (6-gingerol treatmemt)
PA0007		hypothetical protein	-1.0	14.0	NA	1.1
PA0026	*plcB*	hypothetical protein	1.0	5.9	NA	-1.8
PA0027		hypothetical protein	1.0	5.7	NA	1.0
PA0028		hypothetical protein	NA	8.2	NA	1.1
PA0052		hypothetical protein	3.0	22.0	6.7	1.4
PA0059	*osmC*	osmotically inducible protein OsmC	2.0	22.0	NA	-1.6
PA0105	*coxB*	cytochrome c oxidase subunit II	NA	2.6	5.7	-1.2
PA0106	*coxA*	cytochrome c oxidase subunit I	NA	3.3	NA	-1.2
PA0107		conserved hypothetical protein	NA	4.9	6.1	-1.3
PA0108		cytochrome c oxidase subunit III	NA	2.8	NA	-2.0
PA0109		hypothetical protein	NA	4.1	NA	-1.3
PA0122		conserved hypothetical protein	25.0	51.0	29.0	-1.5
PA0132		beta-alanine--pyruvate transaminase	NA	4.1	NA	1.1
PA0143	*nuh*	probable nucleoside hydrolase	2.0	5.4	NA	-1.1
PA0144		hypothetical protein	-1.0	28.0	17.0	1.1
PA0158		probable RND efflux transporter	-2.0	2.6	NA	1.2
PA0175		probable chemotaxis protein methyltransferase	NA	4.6	NA	1.0
PA0176		probable chemotaxis transducer	NA	3.9	NA	1.0
PA0178		probable two-component sensor	-2.0	NA	NA	1.0
PA0179		probable two-component response regulator	-2.0	3.7	NA	-1.5
PA0198	*exbB1*	transport protein ExbB	NA	3.7	NA	1.0
PA0263	*hcpA /// hcpB /// hcpC*	secreted protein Hcp	NA	9.4	NA	-1.5
PA0355	*pfpI*	protease PfpI	NA	8.1	NA	1.1
PA0364		probable oxidoreductase	NA	3.0	NA	-1.2
PA0365		hypothetical protein	NA	2.7	NA	-1.1
PA0366		probable aldehyde dehydrogenase	NA	2.5	NA	1.1
PA0399		cystathionine beta-synthase	NA	NA	5.2	1.0
PA0447	*gcdH*	glutaryl-CoA dehydrogenase	NA	NA	7.8	1.0
PA0534		conserved hypothetical protein	NA	9.8	NA	1.0
PA0567		conserved hypothetical protein	1.0	11.0	NA	1.4
PA0572		hypothetical protein	5.0	19.0	7.8	1.0
PA0582	*folB*	dihydroneopterin aldolase	-2.0	NA	NA	1.0
PA0583		hypothetical protein	-2.0	NA	NA	1.1
PA0586		conserved hypothetical protein	NA	4.6	NA	1.1
PA0588		conserved hypothetical protein	NA	NA	7.4	1.0
PA0744		probable enoyl-CoA hydratase/isomerase	NA	NA	7.0	1.1
PA0745		probable enoyl-CoA hydratase/isomerase	NA	NA	8.2	-1.2
PA0844	*plcH*	hemolytic phospholipase C precursor	2.0	NA	NA	1.1
PA0850		hypothetical protein	-2.0	NA	NA	1.0
PA0852	*cbpD*	chitin-binding protein CbpD precursor	19.0	94.0	18.0	-1.4
PA0855		hypothetical protein	NA	3.0	NA	1.0
PA0981		hypothetical protein	2.0	NA	NA	1.0
PA0996	*pqsA*	probable coenzyme A ligase	295.0	42.0	NA	-1.4
PA0997	*pqsB*	hypothetical protein	96.0	200.0	5.9	-3.0
PA0998	*pqsC*	hypothetical protein	390.0	200.0	7.2	-3.4
PA0999	*pqsD*	3-oxoacyl-[acyl-carrier-protein] synthase III	77.0	45.0	5.1	-2.3
PA1000	*pqsE*	hypothetical protein	152.0	44.0	NA	-4.7
PA1001	*phnA*	anthranilate synthase component I	63.0	290.0	NA	-3.5
PA1002	*phnB*	anthranilate synthase component II	30.0	28.0	NA	-2.3
PA1003	*mvfR*	probable transcriptional regulator	2.0	78.0	NA	-1.2
PA1130	*rhlC*	hypothetical protein	16.0	16.0	7.4	1.0
PA1131		probable MFS transporter	11.0	7.9	5.4	1.0
PA1152		hypothetical protein	-8.0	NA	NA	-1.4
PA1168		hypothetical protein	12.0	NA	NA	1.3
PA1173	*napB*	cytochrome c-type protein NapB precursor	NA	4.1	NA	-1.3
PA1175	*napD*	NapD protein of periplasmic nitrate reductase	NA	3.8	NA	-1.4
PA1176	*napF*	ferredoxin protein NapF	NA	5.8	NA	1.1
PA1177	*napE*	periplasmic nitrate reductase protein NapE	NA	3.6	NA	1.3
PA1196		probable transcriptional regulator	-1.0	NA	NA	-1.1
PA1212		probable MFS transporter	NA	NA	6.9	1.0
PA1214		hypothetical protein	NA	NA	5.0	1.2
PA1215		hypothetical protein	NA	55.0	NA	1.0
PA1216		hypothetical protein	NA	120.0	NA	1.0
PA1217		probable 2-isopropylmalate synthase	NA	380.0	30.0	1.2
PA1218		hypothetical protein	NA	160.0	NA	1.1
PA1219		hypothetical protein	NA	NA	5.3	1.1
PA1221		hypothetical protein	NA	11.0	NA	1.0
PA1245		hypothetical protein	NA	11.0	NA	-1.3
PA1246	*aprD*	alkaline protease secretion protein AprD	67.0	6.6	6.3	-1.3
PA1247	*aprE*	alkaline protease secretion protein AprE	13.0	9.1	NA	1.1
PA1248	*aprF*	alkaline protease secretion protein AprF	150.0	5.2	NA	-1.4
PA1249	*aprA*	alkaline metalloproteinase precursor	39.0	22.0	6.9	1.1
PA1250	*aprI*	alkaline proteinase inhibitor AprI	12.0	24.0	12.0	-1.5

PA1289		hypothetical protein	NA	2.6	NA	-1.1
PA1317	*cyoA*	cytochrome o ubiquinol oxidase subunit II	NA	15.0	NA	-1.4
PA1318	*cyoB*	cytochrome o ubiquinol oxidase subunit I	NA	16.0	5.7	-1.2
PA1319	*cyoC*	cytochrome o ubiquinol oxidase subunit III	NA	7.9	NA	1.0
PA1320	*cyoD*	cytochrome o ubiquinol oxidase subunit IV	NA	9.1	NA	1.1
PA1323		hypothetical protein	3.0	9.6	NA	1.2
PA1324		hypothetical protein	2.0	8.5	NA	-1.2
PA1386		probable ATP-binding component of ABC transporter	-2.0	NA	NA	1.5
PA1404		hypothetical protein	NA	3.8	NA	-1.1
PA1431	*rsaL*	regulatory protein RsaL	150.0	39.0	7.7	1.5
PA1432	*lasI*	autoinducer synthesis protein LasI	5.0	7.7	NA	1.0
PA1656		hypothetical protein	4.0	5.7	NA	1.0
PA1657		conserved hypothetical protein	20.0	24.0	6.6	1.0
PA1658		conserved hypothetical protein	4.0	17.0	NA	-1.4
PA1659		hypothetical protein	13.0	17.0	NA	1.2
PA1660		hypothetical protein	5.0	16.0	NA	-1.3
PA1661		hypothetical protein	NA	4.4	NA	1.1
PA1662		probable ClpA/B-type protease	NA	7.7	NA	1.1
PA1663		probable transcriptional regulator	NA	9.1	NA	1.0
PA1664		hypothetical protein	10.0	22.0	NA	1.0
PA1665		hypothetical protein	3.0	28.0	NA	1.1
PA1666		hypothetical protein	2.0	38.0	NA	1.0
PA1667		hypothetical protein	11.0	12.0	NA	-1.3
PA1668		hypothetical protein	NA	6.3	NA	1.0
PA1669		hypothetical protein	7.0	17.0	NA	1.1
PA1670	*stp1*	serine/threonine phosphoprotein phosphatase Stp1	NA	3.6	NA	1.0
PA1745		hypothetical protein	NA	2.8	NA	1.0
PA1784		hypothetical protein	3.0	18.0	7.3	1.2
PA1869		probable acyl carrier protein	304.0	340.0	79.0	-1.5
PA1871	*lasA*	LasA protease precursor	1274.0	6.3	29.0	1.1
PA1874		hypothetical protein	NA	NA	9.9	-1.1
PA1875		hypothetical protein	1.0	NA	9.1	1.1
PA1877		probable secretion protein	-1.0	NA	NA	1.0
PA1881		probable oxidoreductase	NA	2.8	NA	1.0
PA1888		hypothetical protein	NA	4.3	NA	1.0
PA1891		hypothetical protein	2.0	6.5	NA	1.5
PA1893		hypothetical protein	NA	2.7	NA	1.1
PA1894		hypothetical protein	NA	5.0	25.0	-1.3
PA1895		hypothetical protein	NA	4.2	11.0	-1.1
PA1896		hypothetical protein	NA	3.1	11.0	-1.1
PA1897		hypothetical protein	NA	8.5	33.0	-1.4
PA1901	*phzC1 /// phzC2*	phenazine biosynthesis protein PhzC	80.0	NA	16.0	-36.9
PA1902	*phzD1 /// phzD2*	phenazine biosynthesis protein PhzD	650.0	NA	42.0	-2.0
PA1903	*phzE1 /// phzE2*	phenazine biosynthesis protein PhzE	138.0	NA	26.0	-13.7
PA1904	*phzF1 /// phzF2*	probable phenazine biosynthesis protein	118.0	NA	51.0	-2.1
PA1905	*phzG2*	probable pyridoxamine 5'-phosphate oxidase	116.0	NA	22.0	-9.6
PA1914		conserved hypothetical protein	5.0	700.0	NA	-1.1
PA1921		hypothetical protein	NA	13.0	NA	1.0
PA1927	*metE*	5-methyltetrahydropteroyltriglutamate-homocysteine S-methyltransferase	NA	NA	20.0	-1.3
PA1930		probable chemotaxis transducer	NA	3.8	NA	1.1
PA1939		hypothetical protein	NA	2.9	NA	1.4
PA1999		probable CoA transferase subunit A	NA	NA	32.0	1.1
PA2000		probable CoA transferase subunit B	NA	NA	46.0	1.2
PA2001	*atoB*	acetyl-CoA acetyltransferase	NA	NA	23.0	1.2
PA2014	*gnyB*	probable acyl-CoA carboxyltransferase beta chain	NA	NA	7.5	-1.1
PA2030		hypothetical protein	-1.0	14.0	7.3	1.0
PA2031		hypothetical protein	-2.0	12.0	NA	-1.7
PA2055		probable MFS transporter	11.0	NA	NA	1.0
PA2066		hypothetical protein	NA	12.0	NA	1.2
PA2067		probable hydrolase	NA	19.0	NA	-1.1
PA2068		probable MFS transporter	13.0	150.0	8.6	1.0
PA2069		probable carbamoyl transferase	38.0	110.0	8.8	-1.4
PA2070		hypothetical protein	3.0	NA	NA	1.0
PA2076		probable transcriptional regulator	3.0	4.3	NA	1.2
PA2079		probable amino acid permease	-3.0	NA	NA	1.1
PA2080	*kynU*	hypothetical protein	2.0	4.0	NA	1.1
PA2081	*kynB*	hypothetical protein	NA	3.6	NA	-1.3
PA2082		probable transcriptional regulator	-1.0	NA	NA	1.3
PA2088		hypothetical protein	13.0	NA	NA	1.4
PA2134		hypothetical protein	NA	7.9	NA	1.2
PA2142		probable short-chain dehydrogenase	NA	19.0	NA	-1.3
PA2143		hypothetical protein	NA	51.0	NA	1.2
PA2144	*glgP*	glycogen phosphorylase	NA	15.0	NA	-1.2
PA2146		conserved hypothetical protein	NA	11.0	NA	-1.4
PA2147	*katE*	catalase HPII	NA	35.0	NA	1.0
PA2148		conserved hypothetical protein	NA	3.4	NA	-1.2
PA2151		conserved hypothetical protein	NA	34.0	NA	1.3
PA2152		probable trehalose synthase	NA	6.1	NA	-1.2

PA2153	*glgB*	14-alpha-glucan branching enzyme	NA	16.0	NA	1.0
PA2156		conserved hypothetical protein	NA	17.0	NA	1.1
PA2157		hypothetical protein	NA	2.9	NA	1.3
PA2158		probable alcohol dehydrogenase (Zn-dependent)	NA	26.0	NA	1.1
PA2159		conserved hypothetical protein	NA	10.0	NA	1.0
PA2160		probable glycosyl hydrolase	NA	5.8	NA	1.1
PA2161		hypothetical protein	NA	10.0	NA	1.2
PA2163		hypothetical protein	NA	31.0	NA	1.0
PA2164		probable glycosyl hydrolase	NA	6.5	NA	1.4
PA2165		probable glycogen synthase	NA	6.3	NA	1.3
PA2166		hypothetical protein	NA	17.0	NA	1.0
PA2167		hypothetical protein	NA	4.7	NA	1.5
PA2169		hypothetical protein	NA	5.1	NA	-1.1
PA2170		hypothetical protein	NA	13.0	NA	1.3
PA2171		hypothetical protein	NA	22.0	NA	1.5
PA2172		hypothetical protein	NA	12.0	NA	1.5
PA2173		hypothetical protein	NA	17.0	NA	2.0
PA2176		hypothetical protein	NA	27.0	NA	1.1
PA2180		hypothetical protein	NA	2.7	NA	1.0
PA2190		conserved hypothetical protein	NA	7.5	NA	1.1
PA2192		conserved hypothetical protein	NA	8.4	NA	1.3
PA2193	*hcnA*	hydrogen cyanide synthase HcnA	144.0	88.0	210.0	-1.1
PA2194	*hcnB*	hydrogen cyanide synthase HcnB	87.0	59.0	52.0	1.0
PA2195	*hcnC*	hydrogen cyanide synthase HcnC	46.0	46.0	30.0	-1.2
PA2196		probable transcriptional regulator	NA	NA	5.3	1.1
PA2250	*lpdV*	lipoamide dehydrogenase-Val	NA	NA	7.8	1.3
PA2274		hypothetical protein	NA	11.0	NA	-2.3
PA2300	*chiC*	chitinase	105.0	100.0	5.1	-1.1
PA2302		probable non-ribosomal peptide synthetase	56.0	130.0	27.0	1.8
PA2303		hypothetical protein	346.0	130.0	10.0	1.1
PA2304		hypothetical protein	193.0	29.0	14.0	1.0
PA2305		probable non-ribosomal peptide synthetase	35.0	70.0	28.0	1.1
PA2306		conserved hypothetical protein	NA	NA	13.0	1.0
PA2327		probable permease of ABC transporter	2.0	6.9	NA	-1.3
PA2328		hypothetical protein	NA	7.5	NA	1.2
PA2329		probable ATP-binding component of ABC transporter	NA	18.0	NA	-1.3
PA2330		hypothetical protein	NA	15.0	NA	-1.1
PA2331		hypothetical protein	3.0	20.0	9.4	2.5
PA2345		conserved hypothetical protein	NA	2.6	NA	1.2
PA2365		conserved hypothetical protein	NA	5.9	NA	1.0
PA2366		conserved hypothetical protein	NA	6.9	7.5	-1.1
PA2367		hypothetical protein	NA	6.4	6.3	1.3
PA2368		hypothetical protein	NA	7.5	NA	1.0
PA2370		hypothetical protein	NA	3.5	NA	-1.1
PA2371		probable ClpA/B-type protease	NA	5.0	NA	-1.1
PA2372		hypothetical protein	NA	3.7	NA	1.4
PA2414		L-sorbosone dehydrogenase	2.0	21.0	NA	1.0
PA2415		hypothetical protein	3.0	14.0	NA	1.1
PA2423		hypothetical protein	3.0	13.0	12.0	1.0
PA2433		hypothetical protein	1.0	11.0	NA	1.9
PA2442	*gcvT2*	glycine cleavage system protein T2	NA	3.1	NA	1.1
PA2444	*glyA2*	serine hydroxymethyltransferase	NA	10.0	NA	1.2
PA2445	*gcvP2*	glycine cleavage system protein P2	NA	11.0	NA	1.2
PA2446	*gcvH2*	glycine cleavage system protein H2	NA	18.0	NA	1.2
PA2448		hypothetical protein	NA	12.0	NA	-1.1
PA2512	*antA*	anthranilate dioxygenase large subunit	NA	27.0	NA	1.4
PA2513	*antB*	anthranilate dioxygenase small subunit	NA	13.0	NA	1.2
PA2514	*antC*	anthranilate dioxygenase reductase	NA	3.8	NA	-1.1
PA2552		probable acyl-CoA dehydrogenase	NA	NA	10.0	1.3
PA2553		probable acyl-CoA thiolase	NA	NA	14.0	1.1
PA2554		probable short-chain dehydrogenase	NA	NA	11.0	1.5
PA2555		probable AMP-binding enzyme	NA	NA	6.2	1.1
PA2564		hypothetical protein	6.0	21.0	11.0	1.1
PA2565		hypothetical protein	6.0	14.0	5.5	1.0
PA2566		conserved hypothetical protein	NA	21.0	10.0	-1.4
PA2570	*pa1L*	PA-I galactophilic lectin	90.0	200.0	60.0	1.1
PA2572		probable two-component response regulator	NA	3.3	NA	1.2
PA2573		probable chemotaxis transducer	NA	3.9	NA	1.1
PA2587	*pqsH*	probable FAD-dependent monooxygenase	11.0	15.0	16.0	-1.3
PA2588		probable transcriptional regulator	5.0	46.0	6.9	-1.1
PA2591		probable transcriptional regulator	10.0	42.0	5.7	1.3
PA2592		probable periplasmic spermidine/putrescine-binding protein	14.0	15.0	5.6	1.0
PA2593		hypothetical protein	7.0	29.0	NA	-1.4
PA2594		conserved hypothetical protein	3.0	NA	NA	2.0
PA2629	*purB*	adenylosuccinate lyase	-1.0	NA	NA	-1.3
PA2717	*cpo*	chloroperoxidase precursor	NA	3.4	NA	1.1
PA2747		hypothetical protein	1.0	11.0	NA	-1.1
PA2753		hypothetical protein	-1.0	NA	NA	-1.1

PA2927		hypothetical protein	NA	14.0	NA	1.2
PA2939		probable aminopeptidase	3.0	27.0	16.0	1.0
PA3022		hypothetical protein	NA	4.3	NA	-1.2
PA3032	*snr1*	cytochrome c	NA	9.3	10.0	-1.3
PA3104	*xcpP*	secretion protein XcpP	NA	4.7	NA	-1.8
PA3181		2-keto-3-deoxy-6-phosphogluconate aldolase	NA	3.2	NA	-1.7
PA3182	*pgl*	conserved hypothetical protein	NA	5.0	NA	-3.9
PA3183	*zwf*	glucose-6-phosphate 1-dehydrogenase	NA	4.0	NA	-1.1
PA3188		probable permease of ABC sugar transporter	NA	6.8	NA	-2.0
PA3189		probable permease of ABC sugar transporter	NA	3.0	NA	-1.4
PA3190		probable binding protein component of ABC sugar transporter	NA	4.1	NA	-2.0
PA3194	*edd*	phosphogluconate dehydratase	NA	2.9	NA	-2.3
PA3195	*gapA*	glyceraldehyde 3-phosphate dehydrogenase	NA	5.4	NA	-1.6
PA3274		hypothetical protein	2.0	1.0	NA	1.0
PA3311		conserved hypothetical protein	NA	6.0	NA	1.0
PA3326		probable Clp-family ATP-dependent protease	11.0	19.0	8.7	-1.2
PA3327		probable non-ribosomal peptide synthetase	6.0	20.0	NA	1.2
PA3328		probable FAD-dependent monooxygenase	25.0	47.0	15.0	1.6
PA3329		hypothetical protein	217.0	310.0	55.0	-1.3
PA3330		probable short chain dehydrogenase	159.0	320.0	38.0	1.1
PA3331		cytochrome P450	23.0	62.0	16.0	1.1
PA3332		conserved hypothetical protein	12.0	41.0	15.0	-1.7
PA3333	*fabH2*	3-oxoacyl-[acyl-carrier-protein] synthase III	12.0	64.0	32.0	-1.1
PA3334		probable acyl carrier protein	14.0	69.0	23.0	-1.3
PA3335		hypothetical protein	5.0	29.0	5.1	1.2
PA3336		probable MFS transporter	3.0	24.0	NA	1.2
PA3346		probable two-component response regulator	NA	4.7	NA	1.0
PA3347		hypothetical protein	NA	4.3	NA	1.3
PA3361	*lecB*	hypothetical protein	101.0	68.0	51.0	1.0
PA3369		hypothetical protein	NA	4.8	NA	1.1
PA3370		hypothetical protein	NA	5.6	NA	1.4
PA3371		hypothetical protein	1.0	6.0	NA	1.0
PA3416		probable pyruvate dehydrogenase E1 component beta chain	NA	4.1	NA	1.5
PA3418	*ldh*	leucine dehydrogenase	NA	5.0	NA	1.5
PA3441		probable molybdopterin-binding protein	12.0	NA	NA	1.1
PA3449		conserved hypothetical protein	7.0	NA	NA	1.4
PA3476	*rhlI*	autoinducer synthesis protein RhlL	NA	34.0	NA	-1.2
PA3477	*rhlR*	transcriptional regulator RhlR	11.0	130.0	5.9	-1.6
PA3478	*rhlB*	rhamnosyltransferase chain B	166.0	120.0	37.0	-1.5
PA3479	*rhlA*	rhamnosyltransferase chain A	787.0	200.0	100.0	-3.6
PA3519		hypothetical protein	NA	NA	16.0	-1.1
PA3520		hypothetical protein	24.0	32.0	70.0	-1.1
PA3676		probable RND efflux transporter	NA	5.9	NA	1.2
PA3677		probable RND efflux membrane fusion protein precursor	NA	5.8	NA	1.2
PA3678		probable transcriptional regulator	NA	8.3	NA	-1.2
PA3688		hypothetical protein	NA	3.5	NA	1.3
PA3691		hypothetical protein	2.0	6.3	NA	1.0
PA3692		probable outer membrane protein	2.0	6.9	NA	-1.8
PA3709		probable MFS transporter	NA	NA	5.2	1.3
PA3721		probable transcriptional regulator	NA	NA	5.8	1.1
PA3724	*lasB*	elastase LasB	202.0	240.0	39.0	-1.7
PA3888		probable permease of ABC transporter	NA	3.9	NA	1.0
PA3890		probable permease of ABC transporter	1.0	4.7	NA	-1.1
PA3891		probable ATP-binding component of ABC transporter	NA	8.2	NA	-1.1
PA3904		hypothetical protein	13.0	46.0	18.0	-1.2
PA3905		hypothetical protein	1.0	87.0	NA	1.4
PA3906		hypothetical protein	2.0	71.0	5.8	1.0
PA3907		hypothetical protein	1.0	58.0	NA	1.2
PA3908		hypothetical protein	1.0	55.0	5.9	1.2
PA3923		hypothetical protein	NA	NA	12.0	1.2
PA3939		hypothetical protein	12.0	NA	NA	1.4
PA3986		hypothetical protein	NA	2.8	NA	-1.4
PA3991		hypothetical protein	-2.0	NA	NA	1.3
PA4078		probable nonribosomal peptide synthetase	NA	20.0	7.6	-1.1
PA4085	*cupB2*	probable pili asembly chaperone	-1.0	NA	NA	-1.1
PA4117		probable bacteriophytochrome	NA	4.3	NA	-1.4
PA4129		hypothetical protein	16.0	15.0	14.0	-1.3
PA4130		probable sulfite or nitrite reductase	21.0	1.0	12.0	-2.7
PA4131		probable iron-sulfur protein	13.0	21.0	6.8	-5.7
PA4132		conserved hypothetical protein	3.0	6.4	8.1	-2.2
PA4133		cytochrome c oxidase subunit (cbb3-type)	83.0	37.0	46.0	-1.4
PA4134		hypothetical protein	27.0	21.0	22.0	-5.2
PA4139		hypothetical protein	11.0	3.9	NA	-1.6
PA4141		hypothetical protein	154.0	73.0	25.0	-10.6
PA4142		probable secretion protein	32.0	16.0	9.6	-1.7
PA4171		probable protease	NA	5.1	NA	1.2
PA4172		probable nuclease	NA	14.0	NA	1.3
PA4175	*prpL*	probable endoproteinase Arg-C precursor	3.0	23.0	5.5	1.0

PA4190	*pqsL*	probable FAD-dependent monooxygenase	NA	4.0	NA	1.0
PA4205	*mexG*	hypothetical protein	NA	56.0	8.0	1.0
PA4206	*mexH*	probable RND efflux membrane fusion protein precursor	NA	30.0	5.5	-1.5
PA4207	*mexI*	probable RND efflux transporter	NA	17.0	6.2	-2.1
PA4208	*opmD*	probable outer membrane efflux protein precursor	NA	19.0	6.2	-1.5
PA4209	*phzM*	probable O-methyltransferase	22.0	27.0	14.0	-1.3
PA4210	*phzA1 /// phzA2*	probable phenazine biosynthesis protein	188.0	71.0	NA	1.3
PA4211	*phzB1 /// phzB2*	probable phenazine biosynthesis protein	444.0	220.0	74.0	-1.7
PA4217	*phzS*	probable FAD-dependent monooxygenase	110.0	41.0	19.0	1.0
PA4293		probable two-component sensor	NA	NA	6.6	1.2
PA4294		hypothetical protein	NA	NA	8.6	1.2
PA4296		probable two-component response regulator	NA	5.6	NA	-1.5
PA4297		hypothetical protein	NA	12.0	7.5	1.1
PA4298		hypothetical protein	NA	8.0	6.8	1.0
PA4299		hypothetical protein	NA	7.0	8.9	1.0
PA4300		hypothetical protein	NA	7.8	7.7	-1.1
PA4302		probable type II secretion system protein	NA	7.4	9.6	-1.1
PA4303		hypothetical protein	NA	NA	5.2	1.2
PA4304		probable type II secretion system protein	NA	6.1	6.7	1.2
PA4305		hypothetical protein	NA	5.9	7.3	1.4
PA4306		hypothetical protein	1.0	38.0	28.0	1.3
PA4311		conserved hypothetical protein	NA	2.6	NA	1.1
PA4384		hypothetical protein	NA	4.0	NA	-1.3
PA4496		probable binding protein component of ABC transporter	NA	NA	10.0	1.0
PA4498		probable metallopeptidase	NA	9.1	NA	1.0
PA4590	*pra*	protein activator	-1.0	13.0	NA	-1.2
PA4648		hypothetical protein	NA	17.0	14.0	-1.3
PA4649		hypothetical protein	NA	7.4	NA	1.1
PA4650		hypothetical protein	NA	8.8	NA	1.2
PA4651		probable pili assembly chaperone	NA	15.0	8.8	1.1
PA4652		hypothetical protein	NA	9.6	NA	1.2
PA4677		hypothetical protein	1.0	36.0	15.0	-1.2
PA4703		hypothetical protein	NA	3.5	NA	-1.2
PA4738		conserved hypothetical protein	1.0	11.0	NA	-1.3
PA4739		conserved hypothetical protein	1.0	14.0	NA	-1.3
PA4778		probable transcriptional regulator	3.0	8.6	NA	1.0
PA4869		hypothetical protein	NA	3.8	NA	-1.1
PA4876	*osmE*	osmotically inducible lipoprotein OsmE	NA	4.9	NA	1.1
PA4878		probable transcriptional regulator	NA	NA	8.8	1.0
PA4880		probable bacterioferritin	NA	5.8	NA	-1.3
PA4916		hypothetical protein	NA	6.1	NA	1.0
PA4917		hypothetical protein	NA	7.7	NA	-1.7
PA4925		conserved hypothetical protein	NA	5.7	NA	1.0
PA5027		hypothetical protein	NA	3.2	NA	-1.8
PA5058	*phaC2*	poly(3-hydroxyalkanoic acid) synthase 2	2.0	9.2	NA	1.0
PA5059		probable transcriptional regulator	3.0	9.3	11.0	1.2
PA5061		conserved hypothetical protein	NA	2.6	NA	1.3
PA5162	*rmlD*	dTDP-4-dehydrorhamnose reductase	NA	5.9	NA	1.0
PA5220		hypothetical protein	6.0	26.0	19.0	-1.4
PA5352		conserved hypothetical protein	-1.0	2.9	NA	1.1
PA5353	*glcF*	glycolate oxidase subunit GlcF	-1.0	3.5	NA	-1.3
PA5354	*glcE*	glycolate oxidase subunit GlcE	1.0	3.2	NA	-1.3
PA5355	*glcD*	glycolate oxidase subunit GlcD	NA	3.8	NA	1.1
PA5356	*glcC*	transcriptional regulator GlcC	NA	2.8	NA	-1.2
PA5415	*glyA1 /// glyA2*	serine hydroxymethyltransferase	NA	5.0	NA	-1.4
PA5480		hypothetical protein	1.0	NA	NA	1.4
PA5481		hypothetical protein	1.0	15.0	NA	-1.1
PA5482		hypothetical protein	1.0	18.0	NA	1.1

Hentzer M, *et al* . Attenuation of Pseudomonas aeruginosa virulence by quorum sensing inhibitors. *The EMBO journal* 22, 3803-3815 (2003).

Schuster M, Lostroh CP, Ogi T, Greenberg EP. Identification, timing, and signal specificity of Pseudomonas aeruginosa quorum-controlled genes: a transcriptome analysis. *Journal of bacteriology* 185, 2066-2079 (2003).

Wagner VE, Bushnell D, Passador L, Brooks AI, Iglewski BH. Microarray analysis of Pseudomonas aeruginosa quorum-sensing regulons: effects of growth phase and environment. *Journal of bacteriology* 185, 2080-2095 (2003).

Repressed genes were marked in red color

Gene numbers are annotated for *P. aeruginosa* PAO1.

Supplementary data 3. Expression of QS regulons genes classified by Givskov research group

PA gene	Gene	Description	Regulon	Givskov research group ΔlasIrhlI	ΔlasRrhlR	ΔlasR	ΔrhlR	This study 6-Gingerol treatment
PA0059	*osmC*	osmotically inducible protein OsmC	C	-22.00	-31.80	-44.00	-25.80	-1.6
PA0105	*coxB*	cytochrome c oxidase subunit II	C	-11.50	-13.50	-18.70	-7.90	-1.2
PA0107		conserved hypothetical protein	C	-17.40	-14.10	-18.60	-11.40	-1.3
PA0108	*coIII*	cytochrome c oxidase subunit III	C	-27.80	-8.50	-15.80	-6.30	-2.0
PA0122		conserved hypothetical protein	C	-44.80	-26.10	-7.10	-6.00	-1.5
PA0188		hypothetical protein	C	-8.10	-21.30	-28.20	-8.80	1.3
PA0355	*pfpI*	protease PfpI	C	-11.80	-21.50	-17.70	-11.60	1.1
PA0567		conserved hypothetical protein	C	-249.40	-25.20	-22.10	-9.50	1.4
PA0572		hypothetical protein	A	-10.40	-13.70	-8.20	-1.10	-1.0
PA0737		hypothetical protein	C	-9.40	-7.70	-94.40	-5.80	1.1
PA0843	*plcR*	phospholipase accessory protein PlcR precursor	A	-7.10	-6.20	-25.00	-4.60	-1.0
PA0852	*cpbD*	chitin-binding protein CbpD precursor	C	-63.70	-74.40	-33.20	-8.30	-1.4
PA0990		conserved hypothetical protein	A	-5.70	-19.90	-6.60	-4.40	-1.1
PA0996	*pqsA*	probable coenzyme A ligase	A	-7.00	-20.80	-34.50	3.30	-1.4
PA0997	*pqsB*	hypothetical protein	A	-12.60	-15.40	-59.20	3.10	-3.0
PA0998	*pqsC*	hypothetical protein	A	-8.20	-13.90	-29.90	2.80	-3.4
PA0999	*pqsD*	3-oxoacyl-[acyl-carrier-protein] synthase III	A	-8.20	-9.80	-20.20	2.60	-2.3
PA1000	*pqsE*	hypothetical protein	A	-7.90	-12.00	-28.90	3.60	-4.7
PA1001	*phnA*	anthranilate synthase component I	A	-6.60	-9.70	-20.90	3.20	-3.5
PA1002	*phnB*	anthranilate synthase component II	A	-7.30	-5.20	-6.30	5.50	-2.3
PA1131		probable MFS transporter	B	-5.90	-11.10	-2.90	-6.60	-1.4
PA1168		hypothetical protein	A	-21.50	-19.10	-26.60	-3.90	1.3
PA1190		conserved hypothetical protein	C	-14.60	-12.40	-12.50	-10.40	1.9
PA1242		hypothetical protein	C	-19.60	-21.30	-6.70	-9.20	1.1
PA1249	*aprA*	alkaline metalloproteinase precursor	D	-7.30	-8.40	-4.10	-2.70	1.1
PA1250	*aprI*	alkaline proteinase inhibitor AprI	D	-9.70	-5.50	-3.00	-1.00	-1.5
PA1323		hypothetical protein	C	-25.30	-39.30	-56.50	-15.50	1.2
PA1324		hypothetical protein	C	-22.10	-26.70	-39.20	-17.00	-1.2
PA1431	*rsaL*	regulatory protein RsaL	A	-3619.10	-3441.90	-3038.00	-1.90	1.5
PA1432	*lasI*	autoinducer synthesis protein LasI	A	-97.00	-133.10	-39.00	-1.50	1.0
PA1471		hypothetical protein	C	-7.80	-5.60	-10.30	-9.70	1.3
PA1556		probable cytochrome c oxidase subunit	C	7.90	6.00	7.80	5.30	1.3
PA1625		conserved hypothetical protein	C	-6.60	-9.00	-6.50	-5.50	1.4
PA1657		conserved hypothetical protein	C	-12.70	-15.40	-12.70	-5.20	-1.0
PA1662		probable ClpA/B-type protease	D	-5.70	-9.00	-4.30	-4.40	1.1
PA1664		hypothetical protein	C	-5.20	-11.00	-15.50	-8.70	-1.0
PA1665		hypothetical protein	A	-5.60	-46.90	-15.10	-2.80	1.1
PA1667		hypothetical protein	A	-5.50	-5.10	-5.00	-3.10	-1.3
PA1669		hypothetical protein	B	-28.60	-12.70	-3.80	-10.30	1.1
PA1784		hypothetical protein	A	-9.40	-11.20	-9.90	-1.40	1.2
PA1869		probable acyl carrier protein	C	-137.10	-287.90	-23.60	-25.10	-1.5
PA1870		hypothetical protein	C	-22.00	-12.70	-8.80	-13.80	-1.0
PA1871	*lasA*	LasA protease precursor	C	-105.40	-147.80	-31.30	-7.10	1.1
PA1874		hypothetical protein	A	-8.00	-6.40	-7.90	1.90	-1.1
PA1875		hypothetical protein	A	-34.20	-9.00	-6.70	1.10	1.1
PA1901	*phzC2*	phenazine biosynthesis protein PhzC	C	-21.80	-25.30	-19.10	-51.20	-36.9
PA1902	*phzD2*	phenazine biosynthesis protein PhzD	C	-133.50	-132.00	-97.70	-91.00	-2.0
PA1903	*phzE2*	phenazine biosynthesis protein PhzE	C	-17.20	-24.40	-18.60	-19.70	-13.7
PA1904	*phzF2*	probable phenazine biosynthesis protein	C	-134.00	-61.60	-77.40	-44.70	-2.1
PA1905	*phzG2*	probable pyridoxamine 5'-phosphate oxidase	C	-14.10	-32.70	-20.80	-30.00	-9.6
PA1914		conserved hypothetical protein	A	-92.80	-15.10	-100.00	-1.40	-1.1
PA2021		hypothetical protein	C	-8.60	-12.10	-12.20	-13.20	1.1
PA2046		hypothetical protein	C	-32.60	-22.10	-38.70	-45.50	2.1
PA2067		probable hydrolase	A	-6.00	-5.70	-5.30	-4.20	-1.1
PA2068		probable MFS transporter	C	-8.50	-19.40	-18.50	-25.00	-1.0
PA2069		probable carbamoyl transferase	C	-29.30	-72.90	-26.10	-26.80	-1.4
PA2137		hypothetical protein	C	-11.40	-15.90	-46.40	-71.30	-1.1
PA2139		hypothetical protein	C	-9.50	-10.00	-7.70	-24.30	1.1
PA2141		hypothetical protein	C	-5.80	-45.30	-80.10	-69.00	1.5
PA2142		probable short-chain dehydrogenase	C	-13.40	-16.40	-18.20	-26.80	-1.3
PA2143		hypothetical protein	C	-67.10	-60.10	-191.30	-83.70	1.2
PA2144	*glgP*	glycogen phosphorylase	A	-7.30	-20.80	-11.40	-4.80	-1.2
PA2146		conserved hypothetical protein	C	-6.90	-10.80	-15.50	-9.70	-1.4
PA2148		conserved hypothetical protein	A	-8.10	-8.10	-6.40	-4.00	-1.2
PA2149		hypothetical protein	C	-6.30	-7.00	-39.50	-11.20	-1.3
PA2151		conserved hypothetical protein	C	-39.30	-124.40	-41.20	-30.60	1.3
PA2153	*glgB*	14-alpha-glucan branching enzyme	C	-6.90	-11.30	-19.10	-9.20	-1.0
PA2158		probable alcohol dehydrogenase (Zn-dependent)	C	-65.00	-10.30	-35.90	-52.40	1.1
PA2163		hypothetical protein	C	-8.20	-8.20	-6.90	-5.30	1.0
PA2165		probable glycogen synthase	C	-8.70	-5.40	-6.20	-7.50	1.3
PA2166		hypothetical protein	C	-15.40	-38.50	-24.40	-19.20	-1.0
PA2167		hypothetical protein	C	-8.70	-8.10	-7.10	-15.40	1.5
PA2170		hypothetical protein	C	-8.20	-14.10	-5.40	-6.50	1.3
PA2171		hypothetical protein	C	-27.60	-109.90	-58.60	-22.50	1.5
PA2176		hypothetical protein	C	-19.20	-25.20	-30.00	-12.00	1.1
PA2178		hypothetical protein	D	-11.20	-6.30	-4.30	-4.90	-1.3
PA2184		conserved hypothetical protein	C	-15.50	-13.90	-17.50	-20.80	1.3
PA2190		conserved hypothetical protein	C	-17.00	-14.90	-9.90	-6.90	1.1
PA2193	*hcnA*	hydrogen cyanide synthase HcnA	B	-26.20	-256.60	-3.00	-9.10	-1.1
PA2194	*hcnB*	hydrogen cyanide synthase HcnB	B	-123.10	-43.10	-2.30	-8.30	-1.0

PA2195	*hcnC*	hydrogen cyanide synthase HcnC	B	-20.50	-38.90	-2.70	-6.90	-1.2
PA2300	*chiC*	chitinase	C	-33.80	-66.70	-82.80	-34.00	-1.1
PA2302		probable non-ribosomal peptide synthetase	A	-13.20	-17.50	-10.10	-1.20	1.8
PA2303		hypothetical protein	A	-87.90	-212.80	-5932.00	-1.30	1.1
PA2304		hypothetical protein	A	-18.10	-23.20	-9.20	1.10	1.0
PA2305		probable non-ribosomal peptide synthetase	A	-6.50	-16.70	-8.80	1.10	1.1
PA2414		L-sorbosone dehydrogenase	C	-32.20	-17.60	-16.40	-7.70	1.0
PA2415		hypothetical protein	C	-13.80	-9.30	-9.20	-5.20	1.1
PA2423		hypothetical protein	A	-5.80	-5.90	-7.60	1.60	-1.0
PA2433		hypothetical protein	C	-25.90	-67.30	-70.00	-32.80	1.9
PA2485		hypothetical protein	C	-12.00	-18.00	-15.50	-12.30	-1.0
PA2486		hypothetical protein	C	-8.90	-10.60	-8.50	-5.70	1.2
PA2566		conserved hypothetical protein	A	-9.60	-7.10	-6.30	-1.70	-1.4
PA2570	*pa1L*	PA-I galactophilic lectin	C	-45.40	-49.20	-27.20	-30.30	1.1
PA2587	*pqsH*	probable FAD-dependent monooxygenase	A	-46.10	-36.10	-20.40	1.30	-1.3
PA2588		probable transcriptional regulator	A	-6.70	-34.30	-87.80	-2.40	-1.1
PA2591		probable transcriptional regulator	D	-7.10	-14.80	-2.20	1.10	1.3
PA2592		probable periplasmic spermidine/putrescine-binding protein	D	-6.70	17.90	-3.80	-1.60	-1.0
PA2708		hypothetical protein	C	-5.80	-7.80	-8.70	-12.50	-1.5
PA2747		hypothetical protein	C	-32.60	-37.30	-37.50	-10.30	-1.1
PA2751		conserved hypothetical protein	C	-8.50	-7.00	-10.30	-6.00	1.2
PA2754		conserved hypothetical protein	C	-11.50	-7.30	-9.10	-5.40	-1.2
PA2777		conserved hypothetical protein	C	-8.40	-6.20	-10.50	-8.90	1.0
PA2873		hypothetical protein	D	-13.20	-5.80	-1.60	-1.30	-1.2
PA2937		hypothetical protein	C	-6.90	-8.60	-9.10	-5.40	1.1
PA2939		probable aminopeptidase	A	-39.80	-9.10	-12.70	1.80	-1.0
PA3231		hypothetical protein	C	-33.70	-13.20	-27.70	-10.00	1.1
PA3273		hypothetical protein	C	-13.30	-13.20	-13.20	-27.10	1.1
PA3274		hypothetical protein	C	-45.10	-37.50	-112.50	-17.50	1.0
PA3326		probable Clp-family ATP-dependent protease	D	-12.30	-11.00	-2.40	-4.10	-1.2
PA3327		probable non-ribosomal peptide synthetase	C	-5.40	-12.70	-5.00	-9.80	1.2
PA3328		probable FAD-dependent monooxygenase	B	-6.50	-26.00	-3.20	-12.40	1.6
PA3329		hypothetical protein	C	-54.90	-173.50	-6.10	-69.30	-1.3
PA3330		probable short chain dehydrogenase	B	-62.40	-179.60	-4.40	-19.50	1.1
PA3331		cytochrome P450	B	-19.80	-62.00	-3.80	-23.80	1.1
PA3332		conserved hypothetical protein	B	-10.20	-18.70	-5.00	-17.70	-1.7
PA3333	*fabH2*	3-oxoacyl-[acyl-carrier-protein] synthase III	B	-17.00	-33.40	-4.10	-10.40	-1.1
PA3334		probable acyl carrier protein	C	-42.80	-17.40	-5.10	-11.20	-1.3
PA3335		hypothetical protein	D	-18.40	-9.40	-2.30	-3.90	1.2
PA3336		probable MFS transporter	B	-8.20	-9.30	-4.30	-22.80	1.2
PA3361	*lecB*	hypothetical protein	C	-22.40	-28.90	-13.20	-15.30	1.0
PA3369		hypothetical protein	C	-19.90	-42.20	-36.50	-14.20	1.1
PA3370		hypothetical protein	C	-39.00	-66.40	-87.10	-31.20	1.4
PA3371		hypothetical protein	C	-23.80	-51.70	-69.20	-25.70	1.0
PA3460		probable acetyltransferase	A	-5.00	-6.00	-7.00	-4.70	-1.3
PA3476	*rhlI*	autoinducer synthesis protein RhlL	D	-19.00	-41.40	-2.40	-1.80	-1.2
PA3478	*rhlB*	rhamnosyltransferase chain B	C	-120.20	-122.70	-19.50	-53.00	-1.5
PA3479	*rhlA*	rhamnosyltransferase chain A	C	-320.20	-196.00	-14.50	-55.00	-3.6
PA3520		hypothetical protein	C	-28.30	-18.30	-13.90	-7.40	-1.1
PA3581	*glpF*	glycerol uptake facilitator protein	C	-61.00	-88.90	-19.10	-35.10	1.2
PA3584	*glpD*	glycerol-3-phosphate dehydrogenase	C	-5.70	-20.40	-12.00	-8.40	-1.5
PA3691		hypothetical protein	C	-11.60	-19.20	-28.80	-12.30	1.0
PA3692		probable outer membrane protein	C	-30.70	-16.80	-37.50	-12.30	-1.8
PA3724	*lasB*	elastase LasB	C	-167.20	-224.30	-15.10	-5.20	-1.7
PA3734		hypothetical protein	C	-7.20	-21.30	-6.60	-8.50	-1.3
PA3788		hypothetical protein	A	-9.20	-5.80	-6.10	-4.00	-1.0
PA3819		conserved hypothetical protein	C	-6.30	-7.00	-9.70	-7.10	1.4
PA3888		probable permease of ABC transporter	A	-5.80	-10.20	-10.50	-4.60	1.0
PA3890		probable permease of ABC transporter	C	-17.80	-87.20	-64.70	-31.80	-1.1
PA3904		hypothetical protein	A	-68.30	-40.70	-172.10	-1.00	-1.2
PA3906		hypothetical protein	A	-7.50	-176.50	-9.50	-1.40	-1.0
PA3907		hypothetical protein	A	-5.80	-13.40	-5.20	1.00	1.2
PA3908		hypothetical protein	D	-6.20	-49.80	-4.50	1.10	1.2
PA4078		probable nonribosomal peptide synthetase	A	-5.60	-11.00	-7.40	-4.80	-1.1
PA4130		probable sulfite or nitrite reductase	D	-9.60	-6.20	-3.30	1.50	-2.7
PA4133		cytochrome c oxidase subunit (cbb3-type)	A	-57.40	-13.00	-6.10	1.60	-1.4
PA4134		hypothetical protein	A	-12.00	5.60	-18.00	-1.20	-5.2
PA4139		hypothetical protein	A	-6.10	-10.20	-7.00	-4.20	-1.6
PA4141		hypothetical protein	C	-73.10	-105.00	-27.60	-57.60	-10.6
PA4142		probable secretion protein	C	-27.20	-71.70	-25.60	-26.40	-1.7
PA4143		probable toxin transporter	C	-8.10	-15.30	-7.30	-9.00	1.1
PA4171		probable protease	C	-5.40	-15.50	-13.60	-9.50	1.2
PA4175	*prpL*	probable endoproteinase Arg-C precursor	A	-28.40	-47.10	-30.40	-1.60	1.0
PA4209	*phzM*	probable O-methyltransferase	C	-9.80	-18.80	-11.90	-7.50	-1.3
PA4210	*phzA1*	probable phenazine biosynthesis protein	C	-65.10	-123.90	-79.70	-20.20	1.3
PA4211	*phzB1*	probable phenazine biosynthesis protein	C	-51.00	-61.20	-59.70	-46.10	-1.7
PA4217	*phzS*	probable FAD-dependent monooxygenase	C	-11.40	-12.90	-8.90	-10.90	-1.0
PA4290		probable chemotaxis transducer	C	-7.20	-6.00	-14.20	-15.90	1.2
PA4345		hypothetical protein	C	-10.60	-9.10	-7.70	-6.60	1.6
PA4377		hypothetical protein	A	-10.10	-7.00	-7.80	-3.20	-1.5
PA4677		hypothetical protein	A	-17.00	-40.00	-22.50	-1.10	-1.2
PA4738		conserved hypothetical protein	C	-39.60	-54.40	-58.10	-16.60	-1.3
PA4739		conserved hypothetical protein	C	-34.30	-34.30	-43.80	-16.60	-1.3
PA4876	*osmE*	osmotically inducible lipoprotein OsmE	C	-18.20	-9.10	-10.80	-6.70	1.1

PA4880		probable bacterioferritin	C	-6.00	-29.60	-12.90	-12.90	-1.3
PA5097		probable amino acid permease	D	-9.50	-5.20	-3.40	-4.80	-1.0
PA5099		probable transporter	C	-16.20	-14.70	-40.50	-39.90	1.6
PA5212		hypothetical protein	C	-8.80	-8.70	-12.10	-5.70	1.1
PA5220		hypothetical protein	B	-7.70	-8.90	-4.20	-6.90	-1.4
PA5235	*glpT*	glycerol-3-phosphate transporter	B	-15.20	-32.40	-2.80	-13.90	-1.1
PA5297	*poxB*	pyruvate dehydrogenase (cytochrome)	D	-17.80	-6.00	-3.80	-2.80	-1.0
PA5480		hypothetical protein	B	-14.20	-19.90	-2.50	-6.10	1.4
PA5481		hypothetical protein	C	-45.60	-50.70	-96.60	-22.60	-1.3
PA5482		hypothetical protein	C	-59.60	-43.00	-81.10	-12.20	1.1

Givskov research group

Skindersoe ME, *et al* . Effects of antibiotics on quorum sensing in Pseudomonas aeruginosa. *Antimicrob Agents Ch* 52, 3648-3663 (2008).
Rasmussen TB, *et al* . Identity and effects of quorum-sensing inhibitors produced by Penicillium species. *Microbiology* 151, 1325-1340 (2005).

Gene numbers are annotated for *P. aeruginosa* PAO1.

Supplementary data 4. Primers used in this study

Primer name	Target gene	Sequences (5' -> 3')	Tm (^oC)	GC %	Product size (bp)	Reference
**Specific gene**						
lasA-F	*lasA*	GGA GCG GCT ACT ACA GCA TC	60.01	60.00	206	This study
lasA-R	*lasA*	CTG GCG CAA CTG ATA TTC CT	60.24	50.00		
lasB-F	*lasB*	AAG CCA TCA CCG AAG TCA AG	60.25	50.00	264	This study
lasB-R	*lasB*	GTA GAC CAG TTG GGC GAT GT	60.00	55.00		
lasI-F	*lasI*	CTA CAG CCT GCA GAA CGA CA	60.20	55.00	168	This study
lasI-R	*lasI*	ATC TGG GTC TTG GCA TTG AG	60.07	50.00		
lasR-F	*lasR*	ACG CTC AAG TGG AAA ATT GG	60.11	45.00	247	This study
lasR-R	*lasR*	GTA GAT GGA CGG TTC CCA GA	59.93	55.00		
rhlA-F	*rhlA*	CGA GGT CAA TCA CCT GGT CT	60.11	55.00	208	This study
rhlA-R	*rhlA*	GAC GGT CTC GTT GAG CAG AT	60.42	55.00		
rhlB-F	*rhlB*	GAG CGA CGA ACT GAC CTA CC	59.87	60.00	208	This study
rhlB-R	*rhlB*	GGG AAT CCC GTA CTT CTC GT	60.33	55.00		
rhlR-F	*rhlR*	AGG AAT GAC GGA GGC TTT TT	60.07	45.00	231	This study
rhlR-R	*rhlR*	CCC GTA GTT CTG CAT CTG GT	60.13	55.00		
rhlI-F	*rhlI*	CTC TCT GAA TCG CTG GAA GG	60.09	55.00	240	This study
rhlI-R	*rhlI*	GAC GTC CTT GAG CAG GTA GG	59.87	60.00		
mvfR-F	*mvfR*	AAC CTG GAA ATC GAC CTG TG	59.97	50.00	238	This study
mvfR-R	*mvfR*	TGA AAT CGT CGA GCA GTA CG	60.01	50.00		
pqsC-F	*pqsC*	CTG ATC TGT TCG GCT TCC TC	59.95	55.00	243	This study
pqsC-R	*pqsC*	GGA GAT GTA CTC GCT GCA CA	60.02	55.00		
pqsD-F	*pqsD*	ACC ATG TGA TCT GCC ATC AA	59.93	45.00	243	This study
pqsD-R	*pqsD*	TCA CCT CCT CAG GTT TAC GG	60.10	55.00		
phnB-F	*phnB*	CGT TTC GAT CAG CAT CAC C	60.22	52.63	186	This study
phnB-R	*phnB*	AAT CGA CTC GGG ATG GAA CT	60.85	50.00		
pqsH-F	*pqsH*	ATG TCT ACG CGA CCC TGA AG	60.28	55.00	169	This study
pqsH-R	*pqsH*	AAC TCC TCG AGG TCG TTG TG	60.30	55.00		
phzC1-F	*phzC1*	AGC GGA TTC TCA AGG GCT AT	60.19	50.00	185	This study
phzC1-R	*phzC1*	GTG GGT CGA ACC GAG ATA GA	60.07	55.00		
phzE1-F	*phzE1*	CTG AGT TTC TTC CGC CAG TT	59.47	50.00	206	This study
phzE1-R	*phzE1*	TCG AGG AAT TCC ATG ACC TC	60.01	50.00		
**House keeping gene**						
proC-F	*proC*	GGC GTA TTT CTT CCT GCT GA	60.35	50	236	Savli et al., 2003
proC-R	*proC*	CCT GCT CCA CTA GTG CTT CG	61.12	60		
**Plasmid Construction**						
lasR-F	*lasR*	ACC GAA TTC CGG GAT TCT CGG ACT GCC GTA C (EcoRI)	72.1	58.1	951	This study
lasR-R	*lasR*	CAG AAG CTT GCC GCT TCG GGA TAA GCC (HindIII)	69.5	59.3		

Savli H, *et al* . Expression stability of six housekeeping genes: a proposal for resistance gene quantification studies of Pseudomonas aeruginosa by real-time quantitative RT-PCR. *Journal of Medical Microbiology* 52, 403-408 (2003).
